# Discovery of small molecule mechanistic target of rapamycin inhibitors as anti-aging and anti-cancer therapeutics

**DOI:** 10.3389/fnagi.2022.1048260

**Published:** 2022-12-06

**Authors:** Zofia Chrienova, David Rysanek, Patrik Oleksak, Dorota Stary, Marek Bajda, Milan Reinis, Romana Mikyskova, Ondrej Novotny, Rudolf Andrys, Adam Skarka, Pavla Vasicova, Josef Novak, Martin Valis, Kamil Kuca, Zdenek Hodny, Eugenie Nepovimova

**Affiliations:** ^1^Department of Chemistry, Faculty of Science, University of Hradec Králové, Hradec Králové, Czechia; ^2^Department of Genome Integrity, Institute of Molecular Genetics of the Czech Academy of Sciences, Prague, Czechia; ^3^Department of Physicochemical Drug Analysis, Faculty of Pharmacy, Jagiellonian University Medical College, Kraków, Poland; ^4^Doctoral School of Medical and Health Sciences, Jagiellonian University Medical College, Kraków, Poland; ^5^Laboratory of Immunological and Tumor Models, Institute of Molecular Genetics of the Czech Academy of Sciences, Prague, Czechia; ^6^Department of Neurology, University Hospital Hradec Kralove, Hradec Králové, Czechia; ^7^Faculty of Medicine in Hradec Králové, Charles University in Prague, Hradec Králové, Czechia

**Keywords:** aging, cancer, mTOR, anti-aging therapy, SASP phenotype

## Abstract

To date, the most studied drug in anti-aging research is the mTOR inhibitor – rapamycin. Despite its almost perfect anti-aging profile, rapamycin exerts one significant limitation – inappropriate physicochemical properties. Therefore, we have decided to utilize virtual high-throughput screening and fragment-based design in search of novel mTOR inhibiting scaffolds with suitable physicochemical parameters. Seven lead compounds were selected from the list of obtained hits that were commercially available (**4, 5,** and **7**) or their synthesis was feasible (**1, 2, 3,** and **6**) and evaluated *in vitro* and subsequently *in vivo*. Of all these substances, only compound **3** demonstrated a significant cytotoxic, senolytic, and senomorphic effect on normal and cancerous cells. Further, it has been confirmed that compound **3** is a direct mTORC1 inhibitor. Last but not least, compound **3** was found to exhibit anti-SASP activity concurrently being relatively safe within the test of *in vivo* tolerability. All these outstanding results highlight compound **3** as a scaffold worthy of further investigation.

## Graphical Abstract

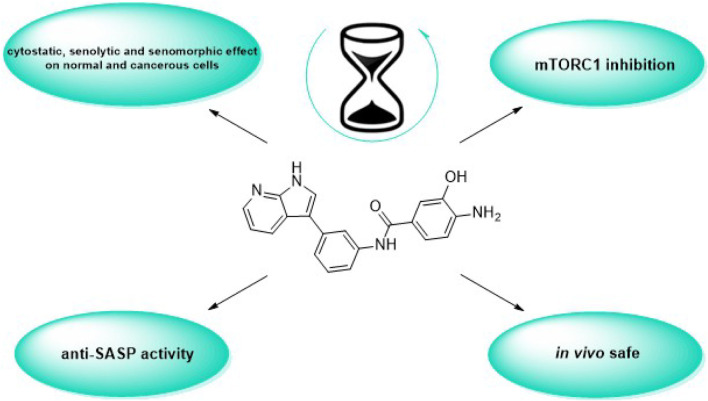

## Introduction

**FIGURE 1:**
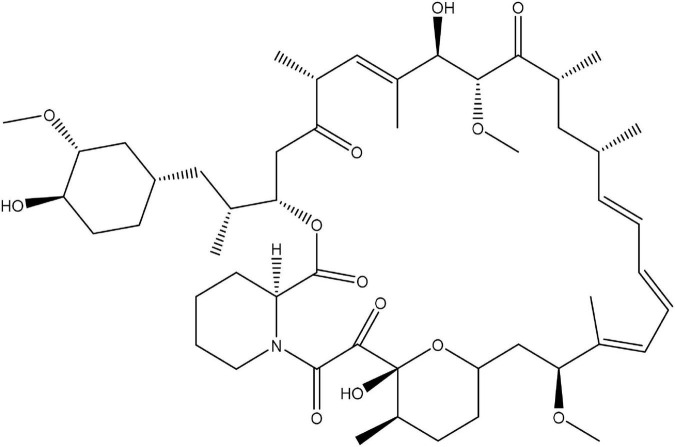
Chemical structure of rapamycin.

According to Einstein’s theory, time is relative and can move slower by increasing the observer’s speed. In the famous twin paradox, this slowing of time enables one identical twin to live longer than the other. Animals treated with rapamycin (Figure 1; Sirolimus), an mTOR inhibitor, also live longer. Of course, that does not mean that rapamycin slows time in the Einsteinian sense. Instead, it figuratively slows biological time by slowing seemingly opposite processes (Blagosklonny, 2018b).

For a better understanding of rapamycin’s mode of action, it is necessary to define the differences between (i) proliferating, (ii) quiescent, and (iii) senescent cells. In proliferating cells, growth factors, hormones, cytokines, and nutrients activate various signaling pathways (including mTOR), which in turn stimulate cellular mass growth coupled with cell cycle progression (Howell and Manning, 2011; Cornu et al., 2013). In quiescent cells, the lack of growth factors and nutrients deactivates the mTOR pathway. Thus, the quiescent cell neither grows nor cycles. Despite such arrest, the cells retain their proliferative potential. Therefore, the re-addition of growth factors may cause reactivation of mTOR signaling, finally resulting in cell mass growth, cell cycle progression, mitosis, and cell proliferation (Leontieva and Blagosklonny, 2014; Terzi et al., 2016). In senescent cells, the cell cycle is blocked, but the mTOR signaling pathway is still active. The senescent phenotype also leads to a loss of the ability to restart proliferation. Thus, in a futile attempt to overcome the cell cycle block, growth-promoting pathways push a cell to become hypertrophic, hyperactive, and hyperfunctional. Such an mTOR-driven process from quiescence to senescence is called geroconversion (Blagosklonny, 2014). The initial hyperfunction is followed by the secondary event – functional decline. For example, initial hyperfunction of osteoclasts (i.e., cells that resorb bones) may result in functional decline – osteoporosis (Blagosklonny, 2006). According to mTOR-centric hyperfunction theory, aging is the sum of age-related diseases, such as osteoporosis, Alzheimer’s disease, diabetes mellitus, cancer, etc. (Blagosklonny, 2021; Chrienova et al., 2021). Therefore, the aim of anti-aging research should not be the treatment of various individual age-related disorders, but rather a novel pharmacological approach to block hyperfunction before it causes the damage.

To date, the most studied drug in anti-aging research is rapamycin. Rapamycin decelerates proliferation while preserving the cell proliferative potential. In that way, rapamycin suppresses both cell growth and geroconversion. By doing so, rapamycin reduces cellular hyperfunction, and delays organ damage as well as aging and age-related diseases (Blagosklonny, 2012). Various *in vivo* experiments on normal mice, yeast, worms, and flies just confirmed the life-prolonging effect of rapamycin. Additionally, rapamycin demonstrated preventative action against age-related changes in rodents, dogs, non-human primates, and humans (Blagosklonny, 2019). Despite the almost perfect anti-aging profile of rapamycin, there is one significant limitation – its physicochemical properties. Taking into account the fact that efficient anti-aging therapy should block geroconversion in all body compartments, it is expectable that such drugs would also achieve therapeutic concentrations in the central nervous system to prevent the development of age-related diseases such as Alzheimer’s disease or Parkinson’s disease. Hence, in recent years, the discovery of small molecules with similar effects as rapamycin, whether as anti-cancer or as the anti-aging treatment, has experienced a relatively notable boom (Zaytseva et al., 2012; Lamming et al., 2013).

Before the development of rapamycin alternatives, the proper biological target needs to be characterized. In this case, it is the mechanistic target of rapamycin (mTOR). mTOR is a large ubiquitously expressed multi-effector serine/threonine kinase that belongs to the phosphatidylinositol 3-kinase (PI3K)-related kinase family (Hoeffer and Klann, 2010). mTOR controls many essential cellular functions such as protein synthesis, energy metabolism, cell size, lysosome biogenesis, transcription, autophagy, actin dynamics, etc. (Bockaert and Marin, 2015). Two structurally and functionally distinct mTOR-containing complexes have been identified. The first, mTOR complex 1 (mTORC1), contains the defining components Raptor (regulatory associated protein of mTOR) and PRAS40 (proline-rich Akt substrate 40 kDa). mTORC1 activity and complex formation are sensitive to rapamycin. mTOR inhibition by rapamycin passes of several steps. Initially, binding of FKBP12-rapamycin to mTORC1 causes subtle conformational change that weakens the mTOR-raptor interaction. In the following step, fast disintegration of already “weakened” mTORC1 occurs (Yip et al., 2010). The second complex, mTORC2, which has been discovered more recently, has Rictor (rapamycin-insensitive companion of TOR), the mammalian stress-activated MAP kinase-interacting protein 1 (mSin1), and Protor-1 and Protor-2 (protein observed with Rictor 1 and 2) as signature components (Laplante and Sabatini, 2012; Costa-Mattioli and Monteggia, 2013). Apart from the active site of mTORC1 that binds ATP, an additional domain should be introduced. The so-called FRB (FKBP12-rapamycin binding) domain is the site of inhibitory interaction between rapamycin and mTOR. In essence, the FRB domain is an allosteric site where the competition between the Raptor (mTOR-stimulating effect) and the complex formed by FKBP12 (FK506 binding protein 12) and rapamycin (inhibitory effect) for binding to the FRB domain occurs (Kim et al., 2002).

Development of drugs that inhibit cell proliferation is generally recognized as an oncology-like task. Thus, unsurprisingly one of the first mTOR inhibitors had been tested against cancer. However, with the passage of time and the discovery of other significant facts, it has been found that by the reduction in the tested concentration of mTOR inhibitors, the given substances could be repurposed as gerosuppressant (Leontieva and Blagosklonny, 2016). Based on this fact, we decided to aim this work not only to the anti-aging potential of novel compounds, but also to anti-cancer.

### Design

In the previous work, we analyzed the binding sites of mTOR and validated docking protocols for easier searching for novel mTOR kinase inhibitors (Stary et al., 2022). In the current study, we continued our efforts aimed at the identification and characterization of novel mTOR inhibitors with suitable physicochemical properties. For this purpose, two different methods were utilized. The first included a virtual high-throughput screening of commercially available compounds from the ZINC database. The latter one consisted of fragment-based design (FBD). Regarding binding site selection, we have employed the ATP binding site for competitive inhibitors search and the FRB domain for allosteric inhibitors investigation. From the list of obtained hits, we have selected those lead compounds that were purchasable or their synthesis was feasible (Table 1). For subsequent experiments, rapamycin and torkinib (PP242) were also purchased as standards with the affinity to FRB and ATP-binding domains, respectively (Tong et al., 2015).

**TABLE 1:** List of selected lead compounds that were forwarded for further investigation.

Compound	Chemical structure	Binding site	Access	Method
1	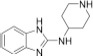	ATP	Synthesized	FBD
2	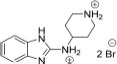	ATP	Synthesized	FBD
3	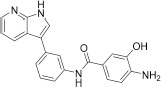	ATP	Synthesized	FBD
4	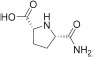	ATP	Purchased	ZINC
5	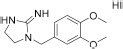	FRB	Purchased	ZINC
6	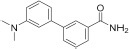	FRB	Synthesized	ZINC
7	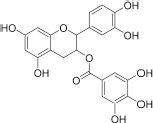	FRB	Purchased	ZINC

## Materials and methods

### *In silico* experiments

#### Computational study

To discover novel potential mTOR kinase inhibitors, virtual high-throughput screening of the ZINC database and fragment-based design approaches have been utilized. Selected ligands were docked into the ATP binding site of mTOR, according to the earlier validated procedure (Stary et al., 2022). Before docking, compounds were prepared with LigPrep module in pH 7.4 ± 0.2 using Epic function (Schrödinger Release 2021-3; Schrödinger, LLC: New York, NY, 2021). The crystal structure of mTOR was downloaded from the PDB database (PDB code: 4JT6). Magnesium ions were added to the active site. Protein was prepared with Protein Preparation Wizard from Schrödinger Suite. Hydrogen atoms were added, and protein was ionized at pH 7.4 ± 0.2. The binding site was defined with Grid Generation module: the grid for ligand docking was set as an internal box with an edge length of 10 Å and a center corresponding to the co-crystallized ligand (PI-103). For each compound, ten poses were obtained. Analogically, compounds proposed as potential rapamycin binding site inhibitors were docked to the FRB domain (PDB code: 4DRJ). The binding site was defined based on the rapamycin position. The development of potential FRB domain ligands was based on the virtual high-throughput screening of the ZINC and Natural Product database. Obtained results were analyzed in PyMOL and Maestro. Physicochemical properties were predicted with the QikProp module from Schrodinger Suite. Based on the scoring function values, binding modes and predicted physicochemical properties of proposed ligands were sorted and analyzed. Finally, seven ligands have been selected for detailed examination and biological evaluation.

#### Molecular dynamics simulation

All-atom systems and input files for molecular dynamics simulations were prepared with CHARMM-GUI online server (Jo et al., 2008; Brooks et al., 2009). The protein-ligand complex was solvated with TIP3P water molecules. Sodium and chloride ions were added (0.15 M NaCl). Equilibration was performed via a one-step protocol implemented in CHARMM-GUI. Molecular dynamics simulation was carried out with NAMD 2.13 at 303.15 K. Timestep was set as 2 fs and the total time was 10 ns. Force field CHARMM36m was used for calculations. The results were analyzed in the VMD program.

### Chemistry

All solvents, reagents, and the final compounds **4**, **5**, and **7** were commercially available (Sigma Aldrich, Prague, Czech Republic; Activate Scientific, Prien, Germany; Penta Chemicals, Prague, Czech Republic; VWR, Stribrna Skalice, Czech Republic; MolPort SIA, Riga, Latvia) and they were used without any further purification.

Reactions were monitored by thin-layer chromatography (TLC) performed on Merck aluminum sheets, silica gel 60 F254 (Darmstadt, Germany). Plates were visualized by UV (254 and 366 nm) or appropriate stain reagent solution. Preparative column chromatography was performed on silica gel 60 (70–230 mesh, 63–200 μm, 60 Å pore size). Flash chromatography was performed on Reveleris^®^Prep purification system (BÜCHI Labortechnik AG, Flawil, Switzerland) using FlashPure BÜCHI Silica 40 μm irregular columns (4–80 g) or FlashPure BÜCHI C18 Silica 40 μm irregular columns (12–24 g). Melting points were acquired on a Büchi M-565 melting point apparatus (BÜCHI Labortechnik AG, Flawil, Switzerland) and were uncorrected.

Nuclear magnetic resonance (NMR) spectra were recorded on Bruker Avance NEO 500 MHz (499.87 MHz for ^1^H and 125.71 MHz for ^13^C). Chemical shifts δ are given in ppm and referenced to the signal center of solvent peaks (DMSO-*d*_6_: δ 2.50 and 39.52 ppm; Chloroform-*d*: δ 7.26 and 77.16 ppm; CD_3_OD: δ 3.31 and 49.0 ppm). Coupling constants are expressed in Hz.

High-resolution mass spectra (HRMS) were obtained by coupled LC-MS system consisting of Dionex UltiMate 3000 analytical LC system (ThermoFisher Scientific, Bremen, Germany) and Q Exactive Plus hybrid quadrupole-orbitrap spectrometer (ThermoFisher Scientific, Bremen, Germany). Heated electro-spray ionization (HESI) was utilized as an ion source (setting: sheath gas flow rate 40, aux gas flow rate 10, sweep gas flow rate 2, spray voltage 3.2 kV, capillary temperature 350°C, aux gas temperature 300°C, S-lens RF level 50). Positive ions were monitored in the range of 100–1,500 m/z, with the resolution set to 140,000. Xcalibur 3.0.63 software (ThermoFisher Scientific, Bremen, Germany) was used to process obtained mass spectra. The non-calibrated purity of the final compounds was determined by UHPLC–MS analysis using Agilent Infinity II 1290 system coupled with 6,470 Series Triple Quadrupole mass spectrometer (electrospray ionization) and UV-VIS spectrophotometer (254 nm) (Agilent Technologies) as detectors. Chromatographic separation was performed on a Zorbax RRHD Eclipse plus C18 column (2.1 mm × 50 mm, 1.8 μm) (Agilent Technologies). Eluents: (A) 0.1% formic acid in water; (B) 0.1% formic acid in acetonitrile.

#### Synthetic procedures and compound characterization

##### Ethyl 4-[(1*H*-benzo[*d*]imidazol-2-yl)amino]piperidine-1-carboxylate (8)

2-Chlorobenzimidazole (500 mg, 3.27 mmol) was combined with ethyl 4-amino-1-piperidine carboxylate (1.13 mL, 6.55 mmol) and heated at 170°C for 5 h. The reaction mixture was cooled to RT and heated CHCl_3_ was added. The resulting solution was extracted with aqueous NaHCO_3_ twice, organic layers were combined, dried over anhydrous Na_2_SO_4_, and evaporated. The residue was added EtOAc, the precipitate was filtered by the fritted glass and rinsed with a small amount of EtOAc. The compound was dried under reduced pressure to give a brown solid.

Yield 643 mg, 68%. Purity: 96%. M.p. 235–237°C. ^1^H NMR (500 MHz, CD_3_OD) δ 7.21–7.17 (m, 2H), 6.98–6.94 (m, 2H), 4.15–4.06 (m, 4H), 3.80 (tt, *J* = 10.6, 4.0 Hz, 1H), 3.09–2.95 (m, 2H), 2.09–2.02 (m, 2H), 1.44 (ddd, *J* = 15.7, 12.5, 4.2 Hz, 2H), 1.26 (*t, J* = 7.1 Hz, 3H). ^13^C NMR (126 MHz, CD_3_OD) δ 157.21, 155.86, 121.29, 112.71, 62.70, 50.85, 43.90, 33.30, and 14.95. MS (ESI) calcd for C_15_H_21_N_4_O_2_ [M+H]^+^ 289.16, found 289.1.

##### *N*-(piperidine-4-yl)-1*H*-benzo[*d*]imidazol-2-amine (1)

The intermediate **8** (100 mg, 0.35 mmol) was dissolved in 47% HBr (1.75 mL) and heated at reflux for 7.5 h. After the reaction was completed, the solvent was removed *in vacuo*, and the residue was purified by silica gel column chromatography (DCM:MeOH:concd NH_4_OH 30:10:1) to give the final product **1** as light yellow solid.

Yield 70 mg, 93%. Purity: 98%. M.p. 227–229°C. ^1^H NMR (500 MHz, CD_3_OD) δ 7.21–7.16 (m, 2H), 6.98–6.94 (m, 2H), 3.77 (tt, *J* = 10.6, 4.0 Hz, 1H), 3.18 (dt, *J* = 13.0, 3.4 Hz, 2H), 2.84 (td, *J* = 12.7, 2.7 Hz, 2H), 2.16–2.09 (m, 2H), 1.60–1.51 (m, 2H). ^13^C NMR (126 MHz, CD_3_OD) δ 155.80, 139.02, 121.31, 112.76, 50.24, 45.41, and 33.05. HRMS (ESI) calcd for C_12_H_17_N_4_ [M+H]^+^ 217.14477, found 217.14430.

##### *N*-(piperidin-4-yl)-1*H*-benzo[*d*]imidazol-2-amine dihydrobromide (2)

Starting from intermediate **8** (300 mg, 1.04 mmol), a similar procedure that was employed for the synthesis of compound **1** gave compound **2** after recrystallization from EtOH as a colorless solid.

Yield 370 mg, 94%. Purity: 99%. M.p. 275–277°C. ^1^H NMR (500 MHz, CD_3_OD) δ 7.45–7.40 (m, 2H), 7.31–7.26 (m, 2H), 4.02 (tt, J = 10.7, 4.0 Hz, 1H), 3.56 (dt, J = 13.2, 3.2 Hz, 2H), 3.26 (td, J = 13.1, 3.1 Hz, 2H), 2.39–2.32 (m, 2H), 2.04–1.93 (m, 2H). ^13^C NMR (126 MHz, CD_3_OD) δ 150.69, 131.20, 124.90, 112.52, 49.67, 44.04, and 29.66. HRMS (ESI) calcd for C_12_H_17_N_4_ [M+H]^+^ 217.14477, found 217.14423.

##### *Tert*-butyl 3-iodo-1*H*-pyrrolo[2,3-*b*]pyridine-1-carboxylate (10)

3-Iodo-1*H*-pyrrolo[2,3-*b*]pyridine (1.0 g, 4.10 mmol) and 4-dimethylaminopyridine (50 mg, 0.42 mmol) were dissolved in dichloromethane (8 mL) and the reaction was cooled to 0°C. A solution of di-*tert*-butyl dicarbonate (1.34 g, 6.15 mmol) in dichloromethane (8 mL) was then added dropwise to the reaction mixture. The mixture was stirred at room temperature for 30 min. After the reaction was completed, it was washed with 0.1 N HCl (20 mL), and the aqueous phase was additionally extracted with DCM (3 × 20 mL). The organic layers were combined, dried with Na_2_SO_4_, and the solvent was evaporated *in vacuo*. The residue was purified by silica gel flash column chromatography (heptane:EtOAc 5–10% gradient) to give **10** as a yellow oil.

Yield 1.31 g, 93%. Purity: 99%. ^1^H NMR (500 MHz, CDCl_3_) δ 8.52 (dd, *J* = 4.8, 1.5 Hz, 1H), 7.79 (s, 1H), 7.71 (dd, *J* = 7.9, 1.6 Hz, 1H), 7.29–7.26 (m, *J* = 7.0, 3.9 Hz, 1H), 1.67 (s, 9H). ^13^C NMR (126 MHz, CDCl_3_) δ 147.65, 146.99, 146.37, 130.87, 129.95, 125.37, 119.37, 84.93, 62.02, and 28.21. MS (ESI) calcd for C_12_H_14_IN_2_O_2_ [M+H]^+^ 345.01, found 345.1.

##### 3-(1*H*-pyrrolo[2,3-*b*]pyridin-3-yl)aniline (11)

Compound **10** (610 mg, 1.77 mmol) and palladium-tetrakis(triphenylphosphine) (62 mg, 0.05 mmol) were dissolved in dry 1,4-dioxane (8.5 mL) under inert atmosphere. Followed by the addition of TEA (2.5 mL, 17 mmol) and pinacolborane (0.38 mL, 2.66 mmol), the reaction was then stirred at 80°C for 3 h. After the starting material was not detected by TLC, the reaction was cooled down to room temperature. Methanol (8.5 mL) was added to the mixture alongside with 3-iodoaniline (0.23 mL, 1.95 mmol) and cesium carbonate (1.44 g, 4.42 mmol), and the reaction was stirred at 100°C overnight. Then, after cooling down to room temperature, the solvents were removed *in vacuo*, and the residue was purified by silica gel column chromatography (DCM:MeOH:TEA 20:1:1%). Still impure product was purified again by silica gel column chromatography (CHCl_3_:TEA 1%) to give **11** as yellow solid.

Yield 305 mg, 82%. Purity: 99%. M.p. 160–162°C. ^1^H NMR (500 MHz, DMSO) δ 11.79 (s, 1H), 8.28–8.22 (m, 2H), 7.70 (*d, J* = 2.5 Hz, 1H), 7.16–7.11 (m, 1H), 7.07 (*t, J* = 7.8 Hz, 1H), 6.97 (*t, J* = 1.9 Hz, 1H), 6.87–6.82 (m, 1H), 6.50–6.45 (m, 1H), 5.08 (s, 2H). ^13^C NMR (126 MHz, DMSO) δ 149.04, 142.74, 135.45, 129.31, 127.58, 123.02, 117.44, 115.77, 115.10, 114.26, 111.98, 111.70, and 45.66. MS (ESI) calcd for C_13_H_12_N_3_ [M+H]^+^ 210.10, found 210.2.

##### 4-{Bis[(*tert*-butoxy) carbonyl]amino}–3-hydroxybenzoic acid (9)

4-Amino-3-hydroxybenzoic acid (1.0 g, 6.52 mmol) was dissolved in the mixture of 1,4-dioxane and water (2:1). The reaction mixture was cooled to 0°C, and TEA (1.37 mL, 9.79 mmol) was added afterward. The solution of di-*tert*-butyl dicarbonate (2.14 g, 9.79 mmol) in 1,4-dioxane was added dropwise to the reaction, and the mixture was stirred at room temperature overnight. The solvents were evaporated, and 1 N HCl (10 mL) was added to the residue. The brown precipitate was filtered, washed with water, and dried *in vacuo*. Product **9** (1.38 g, 60%) was used in the following reaction without further purification.

##### *Tert*-butyl *N*-(*tert*-butoxycarbonyl)-*N*-{2-hydroxy-4-[(3-{1*H*-pyrrolo[2,3-*b*]pyridin-3-yl}phenyl)carbamoyl]phenyl} carbamate (12)

The crude product **9** (500 g, 1.41 mmol) was dissolved in dry DMF (2.5 mL) under inert conditions, and the solution was cooled to 0°C. DIPEA (0.75 mL, 4.2 mmol) and HATU (592 mg, 1.56 mmol) were added to the mixture and stirred for 15 min. The solution of **11** (296 mg, 1.42 mmol) in dry DMF (2.5 mL) was added dropwise to the reaction, and the mixture was stirred at room temperature for 6 h. The reaction was extracted with EtOAc and cold water (2×). The organic layer was dried, and the solvent was evaporated *in vacuo*. The crude product was purified by silica gel flash column chromatography (heptane:EtOAc 25–75% gradient) to give **12** as a yellow solid.

Yield 525 mg, 68%. Purity: 95%. M.p. 142–144°C. ^1^H NMR (500 MHz, CD_3_OD) δ 8.41 (dd, *J* = 8.0, 1.3 Hz, 1H), 8.24 (*d, J* = 4.2 Hz, 1H), 8.15–8.11 (m, 1H), 7.96 (*d, J* = 8.5 Hz, 1H), 7.87 (dd, *J* = 8.6, 2.0 Hz, 1H), 7.81 (*d, J* = 2.0 Hz, 1H), 7.68 (s, 1H), 7.58–7.54 (m, 1H), 7.49–7.39 (m, 2H), 7.20 (dd, *J* = 7.9, 4.8 Hz, 1H), 1.56 (s, 9H), 1.53 (s, *J* = 5.8 Hz, 9H). ^13^C NMR (126 MHz, CD_3_OD) δ 167.26, 154.83, 152.59, 149.87, 143.58, 142.93, 140.30, 136.98, 135.78, 131.36, 130.32, 129.88, 126.54, 124.41, 123.98, 123.14, 120.67, 120.03, 119.82, 117.23, 116.90, 85.21, 81.82, 28.60, and 27.88. MS (ESI) calcd for C_30_H_33_N_4_O_6_ [M+H]^+^ 545.24, found 545.2.

##### *N*-(3-(1*H*-pyrrolo[2,3-*b*]pyridin-3-yl)phenyl)-4-amino-3-hydroxybenzamide (3)

The intermediate **12** (100 mg, 0.18 mmol) was dissolved in DCM (1 mL) and cooled to 0°C. TFA (0.15 mL, 1.8 mmol) was added dropwise to the reaction, and the mixture was stirred at 0°C for 5 h. Afterward, the solvent was evaporated *in vacuo*, and the residue was dissolved in EtOAc. The organic layer was extracted with saturated NaHCO_3_, washed with brine, dried with Na_2_SO_4_, and the solvent was evaporated *in vacuo*. The crude product was purified by C18 silica gel flash column chromatography (water:MeOH 10–95% gradient) to give final product **3** as a brownish solid.

Yield 39 mg, 62%. Purity: 99%. M.p. 173–175°C; ^1^H NMR (500 MHz, CD_3_OD) δ 8.40 (dd, *J* = 8.0, 1.5 Hz, 1H), 8.23 (dd, *J* = 4.8, 1.4 Hz, 1H), 8.09 (*t, J* = 1.6 Hz, 1H), 7.66 (s, 1H), 7.52–7.48 (m, 1H), 7.44–7.31 (m, 4H), 7.18 (dd, *J* = 8.0, 4.8 Hz, 1H), 6.75 (*d, J* = 8.0 Hz, 1H). ^13^C NMR (126 MHz, CD_3_OD) δ 169.45, 149.84, 146.29, 143.51, 142.17, 140.71, 136.84, 130.22, 129.91, 124.82, 124.34, 123.50, 121.02, 120.61, 120.04, 119.78, 117.18, 117.02, 115.03, and 114.95. HRMS (ESI) calcd for C_20_H_17_N_4_O_2_ [M+H]^+^ 345.13460, found 345.13383.

##### 3’-(Dimethylamino)-[1,1’-biphenyl]-3-carboxamide (6)

3-Bromo-*N,N*-dimethylaniline (100 mg, 0.50 mmol) was dissolved in 1,4-dioxane (2.5 mL). [1,1’-Bis(diphenylphosphino)ferrocene]palladium(II) dichloride in complex with dichloromethane (20 mg, 0.03 mmol) and (3-carbamoylphenyl)boronic acid (100 mg, 0.61 mmol) were added subsequently. Finally, a saturated solution of Na_2_CO_3_ (2.5 mL) was added, and the reaction mixture was stirred at 100°C for 4 h. After the reaction was completed, the mixture was filtered through celite (in MeOH), and the solvent was evaporated *in vacuo*. The crude product was purified by silica gel flash column chromatography (heptane:EtOAc 30–60% gradient) to give final product **6** as a brown solid.

Yield 91 mg 75%. Purity: 95%. M.p. 104–106°C. ^1^H NMR (500 MHz, CDCl_3_) δ 8.04 (*t, J* = 1.7 Hz, 1H), 7.78–7.73 (m, 2H), 7.50 (*t, J* = 7.7 Hz, 1H), 7.37–7.30 (m, 1H), 7.00–6.95 (m, 2H), 6.84–6.78 (m, 1H), 6.24 (brs, 1H), 6.03 (brs, 1H), 3.02 (s, 6H). ^13^C NMR (126 MHz, CDCl_3_): δ 169.73, 150.81, 142.80, 141.38, 133.86, 131.05, 129.77, 129.07, 126.47, 126.09, 116.43, 112.54, 111.89, and 41.09. HRMS (ESI) calcd for C_15_H_17_N_2_O [M+H]^+^ 241.13354, found 241.13310.

### Physicochemical analysis

#### Molecular weight, topological polar surface area, hydrogen-bond donors, and central nervous system multiparameter optimization calculations

ACDLAbs PhysChem Suite 14.0 software (Advanced Chemistry Development, Inc., Toronto, ON, Canada) was used for the calculation of Mw, tPSA, and HBD values. The CNS MPO score calculation method was described by Wager et al. (2016).

#### Partition coefficient logP and distribution coefficient logD (pH 7.4)

Before the initiation of the intended experiments, all solvents were mutually saturated at 21°C. 10 nM sodium phosphate buffer (pH 7.4) was used for logD determination. For saturation, large stock bottles containing *n*-octanol and 12% of water/buffer or water/buffer and 12% of *n*-octanol were shaken for 30 mins on a mechanical shaker and subsequently left to stand on the bench overnight until the phases separated. Approximately 0.50 mg of tested substance was added to the test vessels alongside with 1:1 volume ratio of saturated solvents (250 μL). The test vessels were shaken on the mechanical shaker (Multi Reax, Heidolph Instruments, Schwabach, Germany) for 30 min at the highest speed and then centrifuged for phase separation (16.873 × g for 5 min; Eppendorf 5418 Microcentrifuge, Prague, Czech Republic). Both organic and aqueous phases were sampled for LC-MS analysis. UHPLC-DAD-MS (UHPLC Infinity II 1290, detection by DAD and QqQ 6470; Agilent Technologies, CA, USA; separation on Arion C18 polar column 100 × 2.1 mm, 2.1 μm; Chromservis, Czech Republic) was used for determination of the exact concentration of tested substances. The total quantity of substance (in both phases) was used for logP/logD values calculation.

#### Dissociation constants

The spectrophotometric titration was used for dissociation constants’ determination, utilizing spectrophotometer Agilent Cary-60 (Agilent Technologies, CA, USA), pH meter WTW InoLab_IDS Multi 9430 (WTW, Czech Republic), and pH electrode SenTix^®^ Mic (WTW, Czech Republic). Data were analyzed by GraphPadPrism 8.0 software (GraphPad Software, CA, USA). The analyzed samples were prepared by adding 20 μL of compound stock solution (2 mg/mL) and 20 μL of 0.1 M hydrochloric acid into the cuvette with ultrapure water (total volume 3.5 mL). The samples were titrated by 0.1 M sodium hydroxide at 20°C while acquiring absorption spectra (190–500 nm) at different pH values. Absorbance at the chosen wavelength was plotted as a function of pH. The inflection point in the given plot was used for pKa value determination.

#### Solubility

Compounds’ solubility was measured by the NEPHELOstar microplate instrument (BMG Labtech, Offenburg, Germany). Stock solutions were prepared by dissolving the tested compounds in ultrapure water or sodium phosphate buffer (pH 7.4) to obtain a concentration of 160 and 1,000 μg/mL. Each stock solution was sonicated at full power by Hielscher UP100H needle ultrasonic processor (Teltow, Germany) before loading into NEPHELOstar instrument injector A. Ultrapure water or sodium phosphate buffer was loaded into injector B. Compound was diluted in 48 wells in 0–160 or 0–1,000 μg/mL range and subsequently shook for 30 s. Each well was scanned with 80% laser power in matrix 3 × 3, beam width 2 mm, and obtained data were evaluated in GraphPad Prism 7.03 (GraphPad Software, San Diego, CA, USA) using segmental linear regression.

### *In vitro* evaluation

#### Cell cultures

All cell lines used in the study were obtained from the American Type Culture Collection (ATCC, Manassas, VA, USA). Human telomerase-immortalized retinal pigment epithelial cells RPE-1, glioblastoma U-87 MG, glioblastoma U373MG, glioblastoma T98G, glioblastoma A-172, breast adenocarcinoma MDA-MB-231, prostate carcinoma DU-145, and non-small cell lung cancer NCI-H1299 cell lines were cultured in Dulbecco’s modified Eagle’s medium (DMEM, Thermo Fisher Scientific, Waltham, MA, USA) with high glucose (4.5 g/L). Primary human skin BJ fibroblasts were cultured in Dulbecco’s modified Eagle’s medium (DMEM, Thermo Fisher Scientific, Waltham, MA, USA) with low glucose (1 g/L). Both cell culture media were supplemented with 10% fetal bovine serum (FBS, Gibco/Thermo Fisher Scientific) and non-essential amino acids (NEAA), 100 units/mL of penicillin, and 100 μg/mL of streptomycin (Sigma-Aldrich). Cells were kept at 37°C under a 5% CO_2_ atmosphere and 95% humidity.

#### Antibodies

For immunoblotting, the following primary and secondary antibodies were used: rabbit polyclonal p70 S6 kinase (#9202), rabbit monoclonal phospho-p70 S6 kinase (Thr389) (#9234), rabbit monoclonal AKT kinase (#4691), rabbit monoclonal phospho-AKT kinase (Ser473) (#9234) obtained from Cell Signaling Technology, Inc., Danvers MA, USA; p21waf1 (sc-56335) obtained from Santa Cruz Biotechnology, Inc., Dallas, TX, USA and mouse monoclonal anti-GAPDH (GTX3066) purchased from GeneTex, Inc., Irvine, CA, USA; goat anti-mouse (170-6516) or anti-rabbit IgG (H + L)-HRP conjugate (170-6515) purchased from BioRad, Hercules, CA, USA.

#### Induction of cellular senescence

To obtain ionizing radiation (IR)-induced senescent cells, proliferating RPE-1 cells were exposed to a single dose of X-rays (20 Gy) using Pantak HF160 (Gulmay, Surrey, UK) X-ray instrument equipped with Pantak Seifert HF320 generator, MXR-161 X-ray tube (Comet AG, Flamatt, Switzerland), and an aluminum filter using current 1–10 mA. After irradiation, the cells were cultured for an additional 7 days until the development of the senescent phenotype (Salovska et al., 2022). To induce docetaxel-induced senescence (DIS), BJ cells were exposed to 5 nM docetaxel for 7 days, as described previously (Sapega et al., 2018). To prepare temozolomide (TMZ)-induced senescence, U87 cells were exposed to 100 μM TMZ for 14 days.

##### Senescence-associated beta-galactosidase activity

The development of cellular senescence was followed by the determination of senescence-associated beta-galactosidase activity (Dimri et al., 1995). Cells were fixed with 0.5% glutaraldehyde at room temperature for 15 min. Cells were then washed twice with 1 mM MgCl_2_/PBS and incubated with X-Gal staining solution at 37°C for 3 h. The staining was terminated by three consecutive washes with ddH_2_O. Finally, the cells were let dry, mounted with Mowiol containing DAPI, and imaged on the Leica DM6000 fluorescent microscope using the HC PLAN APO 20×/0.70 DRY PH2 objective and color CCD camera Leica DFC490 (Leica Microsystems GmbH, Wetzlar, Germany).

##### Detection of DNA replication by 5-ethynyl-2’-deoxyuridine incorporation assay

Cells were incubated with 10 μM 5-ethynyl-2’-deoxyuridine (EdU) for 24 h and fixed with 4% formaldehyde at room temperature for 15 min. To visualize EdU incorporation, click chemistry was performed with Click-iT EdU Cell Proliferation Kit with Alexa Fluor™ 647 dye (Thermo Fisher Scientific, Waltham, MA, USA) according to the manufacturer’s instructions. The stained cells were acquired by high-content imaging using an inverted wide-field microscope (Olympus IX81) equipped with UPLXAPO 20×/0.8 DRY CORR; FWD 0.6; CG 0–2 objective and sCMOS camera Hamamatsu ORCA-Flash4.0 LT + 90, 6.5 μm pixel.

#### Cell proliferation assays

Cell proliferation was followed by the crystal violet (Feoktistova et al., 2016), resazurin assays (Anoopkumar-Dukie et al., 2005), and time-lapse microscopy.

Twenty-four hours before treatment, cells were plated in 96-well plates at densities of 2,850 cells per well for senescent RPE-1 (IR-RPE-1), 3,350 cells per well for proliferating RPE-1 and senescent BJ (DIS-BJ), and 5,000 cells per well for U373, T98, A172, U87, TMZ-U87, and proliferating BJ cells.

##### Crystal violet assay

For the crystal violet assay, the cells were washed twice with 150 μL PBS and stained with 30 μL 0.5% (w/v) crystal violet in 20% methanol for 15 mins. Next, the plates were washed five times with ddH_2_O and left to dry overnight. Crystal violet was solubilized with 75 μL 0.2% Triton X-100 (Sigma) in PBS for 15 mins. The absorbance was read at 595 nm using a microplate reader (Envision, PerkinElmer, Waltham, MA, USA). The absorbance of crystal violet in treated cells was expressed as a percentage of absorbance in untreated cells. Each condition was measured at least in triplicate.

##### Resazurin assay

For the resazurin assay, 200 μL of the culture medium of each well was exchanged for 100 μL of resazurin (stock solution 30 mg/mL; Sigma, St. Louis, MO, USA) diluted 10 times in the culture medium, and the cells were incubated at 37°C for 1–3 h. Reading of fluorescence was done using a microplate reader (PerkinElmer, Waltham, MA, USA). Absolute values of fluorescence were related to the values of untreated cells. Each condition was measured at least in triplicate.

##### Time-lapse microscopy

For auto-monitoring of cells, Incucyte SX1 Live-Cell Analysis System (Sartorius, Göttingen, Germany), located in an incubator maintained at 37°C under a 5% CO_2_ atmosphere and 95% humidity, was utilized. The images (1,408 × 1,040 pixels at 1.24 microns/pixel) were acquired using a 10× objective lens in phase contrast, red fluorescence (excitation: 585 ± 20 nm, emission: 635 [625, 705] nm, acquisition time: 100 ms) channels every 60 min during 72 h. The first image was acquired 30 min after adding compounds. The images were processed to measure cell confluence using Incucyte software (version 2022A). Annexin V – Dy647, Apronex (diluted 1:1,000) was used for apoptotic cell staining.

#### Determination of proinflammatory cytokines

To determine the secretion of selected inflammatory cytokines into culture media, cell cultures were exposed to tested compounds (50 μM) for 24 h. Then the culture medium was removed, cells were washed three times with 1 mL PBS, and a fresh medium without FBS was added for 24 h. Detection of cytokines in conditioned culture media was done by Human Inflammation 11-Plex Panel: IFNγ, IL-1α, IL-1β, IL-6, IL-8, IL-10, IL-12p70, IL-27, IP-10, MCP-1, and TNFα (AimPlex Biosciences, Inc.) using a BD FACSVerseTM flow cytometer (BD Biosciences, Franklin Lakes, NJ, USA) according to manufacturer’s protocol. Data were analyzed using FlowJo 10 software (FlowJo LLC, Ashland, OR, USA).

#### SDS-PAGE and immunoblotting

Cells were harvested 1 and 5 h after treatment. Cells were washed with PBS, harvested into SDS sample lysis buffer (SLB; 2% SDS, 63 mM Tris–HCl, pH 6.8, and 10% glycerol), sonicated, and centrifuged. Protein concentration was determined by BCA assay (Pierce Biotechnology, Rockford, IL, USA); samples were adjusted to equal protein amount (40 μg) with SLB containing dithiothreitol (1% final conc.) and bromophenol blue (0.02% final conc.), and separated by SDS–PAGE in Tris-glycine-SDS buffer (BioRad, 1610772). Proteins were electrotransferred onto a nitrocellulose membrane (0.45 μm NC, Amersham™, GE Healthcare Life Sciences) using wet transfer in Tris-glycine buffer (BioRad, 1810704) with 10% methanol (Sigma, 59304). After blocking with 5% non-fat milk in PBS/Tween-20, proteins were detected using specific antibodies and horseradish peroxidase (HRP)-conjugated secondary antibodies. Peroxidase activity was detected by ECL detection reagents (Thermo Fisher Scientific, Waltham, MA, USA).

#### *In vitro* mechanistic target of rapamycin kinase enzyme assay

The mTOR enzyme assay was used to determine the mTOR kinase activity of compound **3**. Enzymatic reaction mixture was prepared as follows: mTOR enzyme (10 μg/mL, Invitrogen), ATP (10 μM), p70S6K peptide (10 μg/mL, SignalChem), and kinase buffer (50 mM HEPES, pH 7.5, 1 mM EGTA, 3 mM MnCl_2_, 10 mM MgCl_2_, 2 mM DTT, and 0.01% Tween-20). The reaction was performed in black half-area 96-wells at room temperature for 1 h, then stopped by adding ADP detection buffer (Transcreener ADP^2^ FI Assay, BellBrook labs) and incubated at room temperature for 1 h. Compound concentration was 10, 20, 40, 80, and 100 μM. The final DMSO concentration was 1%. PHERAstar microplate reader was used for measuring the fluorescence intensity (excitation 575 nm, emission 620 nm).

### *In vivo* testing

#### Mice

C57BL/6 (B6) male mice, 8–12 weeks old, were obtained from the Institute of Molecular Genetics, Prague, Czech Republic. Experimental protocols were approved by the Institutional Animal Care Committee of the Institute of Molecular Genetics, Prague.

#### *In vivo* tolerability study

To analyze the tolerance of the new compound **3** and torkinib (as a control), the compounds were perorally administered at increasing doses (three times on days 0, 3, and 5). Compounds were dissolved in 20 μL DMSO and completed into 200 μL with corn oil.

During the experiments, mice behavior and possible apparent signs of toxicity were monitored daily. The body weights were determined on days 0, 4, and 8. On day 8, blood (25 μL) was withdrawn using a capillary pipette containing anticoagulant (heparin) from the retroorbital sinus into EDTA-containing tubes. Basic hematological parameters were measured using the Mindray BC-5300 Vet. Mice were then killed, autopsied, and abdominal organs (spleen, liver, and kidney) were removed for visual examination and imaging by a digital camera. Single-cell suspensions from the spleens were prepared by homogenization through a cell strainer (70 μm; BD Biosciences, San Jose, CA, USA). Erythrocytes were osmotically lysed using ammonium chloride-potassium lysis buffer, and the cell suspensions were washed three times in the RPMI-1640 medium and used for further analysis by flow cytometry for selected cell populations.

#### Spleen cell analysis by flow cytometry

The cellularity of selected spleen cell populations was analyzed by flow cytometry. Cell suspensions were washed and preincubated with anti-CD16/CD32 (2.4G2) antibody to minimize non-specific binding at 4°C for 15 min following the washing step and incubation with labeled primary antibody at 4°C for 30 min. Relevant isotype controls of irrelevant specificity were used. FACS buffer (PBS, 1% FBS, 0.1% NaN_3_) was used for all washing steps and analysis. The following antibodies were used for FACS analyses: BV421 anti-mouse CD45 (30-F11), PE anti-mouse CD8α (53-6.7), BV711 anti-mouse CD4 (RM4-5), APC anti-mouse Ly-6g/Ly-6C (Gr-1) (RB6-8C5), BV711 anti-mouse CD11b (M1/70). FACS analysis was performed using a flow cytometer Symphony (BD Biosciences) and analyzed using FlowJo 10 software (FlowJo LLC, Ashland, OR, USA).

#### Histopathological analysis

Spleen, kidney, and liver tissue samples from the control and treated animals were fixed in 4% formaldehyde and embedded into paraffin. The analysis and evaluation were performed at the Czech Centre for Phenogenomics, Institute of Molecular Genetics of the CAS, Vestec, using Leica ASP6025 automatic vacuum tissue processor for tissue processing, Leica EG1150 H + C embedding station for embedding, and Leica RM2255 rotary microtome for sectioning (2 μm sections). Standard haematoxylin and eosin (HE) descriptive histopathological staining was done using Leica ST5020 + CV5030 stainer and coverslipped.

#### Data processing and statistical analysis

Graphs were generated using GraphPad Prism 5.04 (GraphPad Software, La Jolla, CA, USA). The data are expressed as the mean ± S.D. from three independent experiments. Statistical differences for two groups were analyzed by Student’s *t*-test, ^****^p < 0.0001; ^***^p < 0.001; ^**^p < 0.01; *p < 0.05. Quantitative analysis of immunoblots was done by Image J 1.48v program with GAPDH as the internal control.

## Results and discussion

### Computational study

#### Ligands of adenosine triphosphate binding site

For the given computational study, two different methods have been utilized – fragment-based design and virtual high-throughput screening. Compounds were docked to the ATP binding site of mTOR (PDB code: 4JT6), according to the fully validated procedure (Stary et al., 2022). Further, their scoring function values, binding modes, and physicochemical properties were analyzed. Fragment-based design approach allowed us to obtain promising ligands, of which compound **1** (a benzimidazole derivative), its salt form – compound **2**, and ligand **3** with azaindole fragment (Table 1) have been selected for biological evaluation. Within virtual screening of a part of the ZINC database, a series of small-molecule ligands with interesting binding modes in ATP catalytic cleft has been discovered, of which compound **4** has been selected for *in vitro* evaluation (Table 1).

**FIGURE 2:**
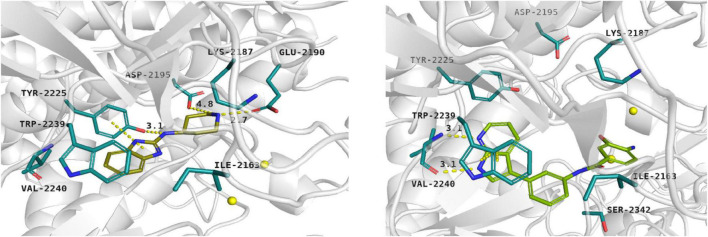
Binding mode of compound **1 (left panel)** and compound **3 (right panel)** within the ATP binding site of mTOR. Magnesium ions – yellow spheres. Selected interactions between ligands and residues colored as yellow dashes.

Docking results showed that compound **1** exerts the following binding mode: benzimidazole ring occupied the same place as the adenine ring of ATP concurrently forming π–π stacking interaction with the side chain of Tyr2225 (Figure 2, left panel). Amine linker of compound **1** coupled with the hydroxy group of Tyr2225 by a hydrogen bond. The ionized amine group of the piperidine ring interacted with polar, negatively charged amino acids, such as Glu2190 and Asp2195 (Figure 2, left panel). Additionally, compound **1** demonstrated a less favorable score value than reference compound torkinib (−6.234 and −8.137, respectively).

Among presented compounds, ligand **3** obtained a promising binding mode as well as a docking score value (−9.580), which was more beneficial compared to that of torkinib. The top-ranked pose of compound **3** within the mTOR kinase active site is presented in Figure 2 (right panel). Herein, the azaindole fragment mimicked the adenine ring of ATP. Compared with ligand **1**, compound **3** interacted with the main chain of Val2240 via hydrogen bonds, thus significantly stabilizing the ligand-protein complex. Mentioned heterocyclic moiety was able to interact with Trp2239 by *π–π* stacking interaction as well as with Tyr2225 via weak aromatic ring interaction. Benzamide scaffold of compound **3** was docked into a site of ribose- and phosphate-moieties binding. The 10 ns molecular dynamics (MD) simulation showed that the overall binding mode of ligand **3** was stable. Furthermore, it was observed that hydrogen bonds between azaindole moiety and Val2240 were also present in the course of the MD run. Initially, a hydrogen bond between the Val2240 oxygen atom and azaindole nitrogen atom was broken, however, this interaction was rapidly renewed (Supplementary Figure 1). Most changes in the position of compound **3** were observed for the benzamide fragment. Such finding suggests that interactions with Val2240 are crucial for binding ligand **3** into the active site of mTOR.

*In silico* studies revealed that ligand **4** interacted with the ATP binding site in the site of phosphate group binding. Compound **4** obtained a beneficial score: −9.068. In addition, it formed salt bridges with Glu2190, Asp2357, and ionic interactions with magnesium ions as well (not shown).

#### Ligands of FKBP12-rapamycin binding domain

Searching for potential mTOR inhibitors acting by allosteric modulation was carried out by means of virtual high-throughput screening of ZINC and natural product database (NPDB). For biological evaluation, three potential allosteric inhibitors have been selected: compounds **5** and **6**, which present a less complex structure than rapamycin, and epicatechin gallate (**7**) from NPDB.

**FIGURE 3:**
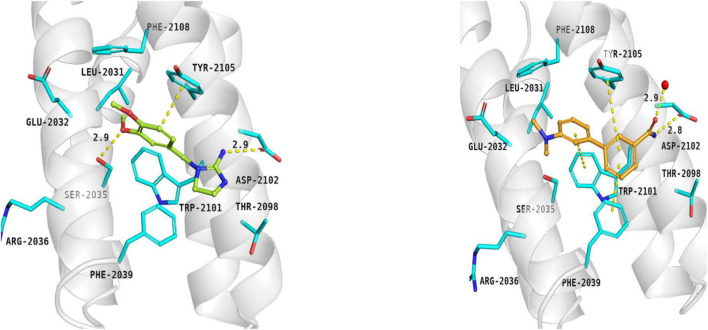
Binding modes of compound **5 (left panel)** and compound **6 (right panel)** within the rapamycin binding site of mTOR. FRB domain – grey cartoon, water ions – red spheres. Selected interactions between ligands and residues colored as yellow dashes.

Compounds **5** and **6** were located in the pocket, occupied by the triene fragment of rapamycin in its complex with mTOR, and formed specific interactions with the FRB domain of the enzyme (Figure 3; compound **5**–left panel; compound **6**–right panel). The methoxy group at benzylimidazolidine scaffold of compound **5** was noticed to form hydrogen bond with Ser2035. On the other hand, phenyl group was able to form aromatic interactions with the side chain of Tyr2105. Additionally, imino-imidazolidine fragment of compound **5** interacted with Asp2102 residue via the salt bridge.

The binding mode of biphenyl derivative **6** was stabilized through π–π interactions with Trp2101, Tyr2105, and Phe2039. Amide substituent formed the hydrogen bond with Asp2102 residue and water molecule 2226.

Epicatechin gallate (**7**) was selected from the Natural Product Database by virtual high-throughput screening. It has been observed that mentioned ligand occupied the rapamycin binding site and interacted with a water molecule and Asp2101 via hydrogen bonds. Additionally, Phe2039 formed an aromatic ring interaction with dihydroxyphenyl moiety (not shown).

### Chemistry

**SCHEME 1:**
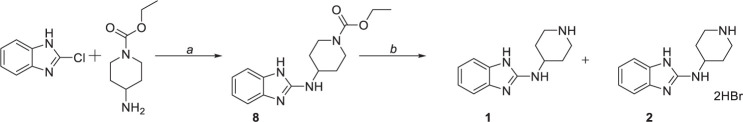
Chemical synthesis of compounds **1** and **2**. Reagents and conditions: (a) 170°C, (b) HBr (47%), reflux.

Compounds **1** and **2** were formed using a two-step reaction process as described in the literature (Kubota et al., 2006). Initially, 2-chlorobenzimidazole was coupled with ethyl 4-amino-1-piperidine carboxylate to give intermediate **8** (Scheme 1a), followed by acidic deprotection to obtain compounds **1** and **2** (Scheme 1b).

**SCHEME 2:**
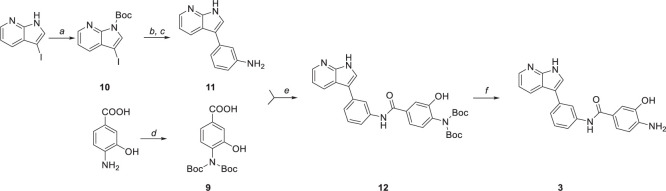
Synthetic route to compound **3**. Reagents and conditions: (a) (Boc)_2_O, DMAP, DCM, 0°C to RT, (b) Pd(PPh_3_)_4_, TEA, HBpin, 1,4-dioxane, 80°C, (c) 3-Iodoaniline, Cs_2_Co_3_, MeOH, 100°C, (d) (Boc)_2_O, TEA, 1,4-dioxane/water (2:1), 0°C to RT, (e) HATU, DIPEA, DMF, 0°C to RT, and (f) TFA, DCM, 0°C.

Compound **3** was prepared in five steps (Scheme 2). Firstly, the amine group of 4-amino-3-hydroxybenzoic acid was protected by di-*tert*-butyl dicarbonate (Scheme 2d) to yield intermediate **9**. Similarly, *N*-Boc protection was performed to obtain intermediate **10** (Scheme 2a) that was subsequently used in the one-pot Masuda borylation – Suzuki coupling sequence. In the first step of the sequence (Scheme 2b), the *N*-protected iodide **10** was left to react with pinacolborane, palladium-tetrakis(triphenylphosphine), and triethylamine as a base in 1,4-dioxane to obtain desired boronic ester. Afterward, methanol and commercially available halide were added to the reaction mixture, followed by cesium carbonate to enable the Suzuki coupling (Scheme 2c). Concurrently, Boc protective group was cleaved by carbonate solution; thus, no additional deprotection step was needed. Obtained intermediate **11** was left to react with previously synthesized intermediate **9** to yield amide **12** followed by the final *N*-Boc acidic deprotection to give final product **3**.

**SCHEME 3:**
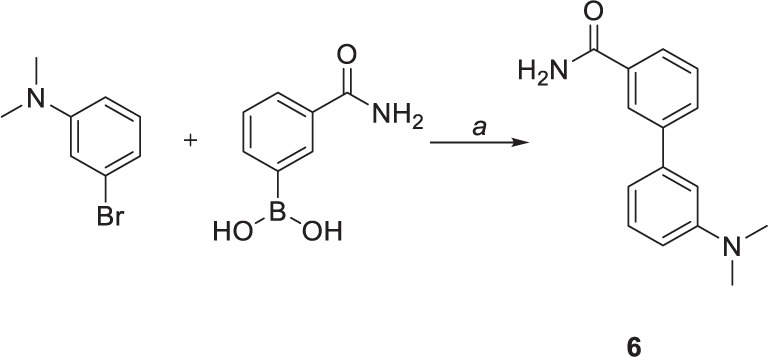
Chemical synthesis of compounds **6**. Reagents and conditions: (a) Pd(dppf)CL_2_.DCM, sat. sol. Na_2_CO_3_, 1,4-dioxane, 100°C.

Suzuki coupling was also performed to obtain compound **6** (Scheme 3). The reaction was carried out by employing Na_2_CO_3_ as base and 1,4-dioxane as solvent. [1,1’-Bis(diphenylphosphino)ferrocene]palladium(II) dichloride in complex with dichloromethane [Pd(dppf)Cl_2_.DCM] was used as a catalyst, affording the coupling product in high yield (75%) after 4 h.

### Physicochemical properties determination

An important factor in the decision-making process when selecting an appropriate drug candidate is pharmacokinetic profile of such candidate. Unfortunately, the excellent activity of rapamycin and its derivatives, so-called rapalogs, is often overshadowed by their poor pharmacokinetic properties. Thus, new potential mTOR inhibitors aim for better absorption, distribution, metabolism, and elimination (ADME) that highly depend on the compound’s physicochemical properties. For this purpose, we used experimental and computational methods to determine the exact physicochemical properties of compounds **1**–**7** as well as selected standards (lipophilicity – logP; distribution coefficient at pH 7.4 – logD; most basic center – pKa; molecular weight – Mw; a number of hydrogen-bond donors – HBDs; and topological polar surface area – tPSA). Subsequently, we applied obtained data to evaluate the central nervous system multiparameter optimization (CNS MPO) score. This score is frequently used for blood-brain-barrier transport estimation of compounds that would indicate suitable pharmacokinetic profile and “all-body compartments” reach.

Spectrophotometric titration of the substances containing chromophore close to the ionization center was used to acquire dissociation constants. pKa values of studied compounds ranged from 3.17 to 7.91 (see Table 2). Experimental logP and logD values were accessed by the adapted shake-flask method in the octanol-aqueous phase. LogP and logD values were equal to (−2.89) – 2.86 and (2.82) – 2.91, respectively. ACDLAbs PhysChemSuite software was used for molecular weight, topological polar surface area, and the number of hydrogen-bond donors calculations. Calculated values ranged 158.16–422.37, 46.33–177.14, and 2–7 for Mw, tPSA, and HBDs, respectively.

**TABLE 2:** Physicochemical properties of selected (**1**–**7**) as well as standard compounds.

Compound	Mw	pKa_1_	pKa_2_	logP	logD	tPSA	HBD	MPO
1	216.28	6.18 ± 0.04	–	1.86 ± 0.07	0.85 ± 0.03	52.74	3	**5.2**
2	376.28	6.08 ± 0.08	–	−1.01 ± 0.01	−0.89 ± 0.11	58.57	5	**4.9**
3	344.37	3.60 ± 0.29	7.91 ± 0.15	2.08 ± 0.07	2.15 ± 0.02	104.03	5	**4.5**
4	158.16	3.17 ± 0.09	7.80 ± 0.11	−2.89 ± 0.16	−2.82 ± 0.22	92.42	4	**4.9**
5	363.20	n/a	n/a	−1.79 ± 0.04	−1.78 ± 0.08	57.58	2	**4.5^a^**
6	240.30	4.80 ± 0.06	–	2.86 ± 0.01	2.91 ± 0.01	46.33	2	**5**
7	422.37	n/a	n/a	1.48 ± 0.03	1.52 ± 0.01	177.14	7	**2.4^a^**
Rapamycin	914.17	10.7 ± 0.21	–	3.97 ± 0.03	4.63 ± 0.04	195.43	3	**0.7**
Torkinib	308.34	3.96 ± 0.02	10.37 ± 0.18	2.65 ± 0.02	2.75 ± 0.06	105.64	4	**3.1**

n/a, not available. ^a^CNS MPO score for compounds with unknown experimental pKa was calculated with T0(pKa) equal to zero.

Central nervous system multiparameter optimization desirability score was calculated as described by Wager et al. (2016), utilizing all collected experimental and calculated data. This scoring tool was set on ranges of desirable property space and linear transformational functions with values between 0 and 1 (defined as T0 for each property). Rating each parameter equally, the collective score ranges from 0 to 6, while a score higher than 4 is desirable (Rankovic, 2015). Inferior CNS MPO score was calculated for epicatechin gallate **7** (2.4), while all other compounds reached the desirable limit with values equal to 4.5–5.2. In combination with acquired *in vitro* results, this promising data was regarded as an essential selective factor of compounds for further in *vivo* testing. Looking at the standard compounds, i.e., rapamycin and torkinib, it is evident that none reached the required CNS MPO score. Therefore, it can be concluded that both compounds cannot reach the “all-body compartments” equally.

For complex characterization of given compounds, solubility in water and buffer with pH 7.4 was assessed (Table 3) by nephelometry assay. The solubility spectrum ranged from miserable to supreme. Compounds **3**, **6**, rapamycin, and torkinib exhibited solubility values in water and buffer lower than 6 μg/mL with logS_H2O_ and logS_pH7.4_ lower than (−4.60). On the other hand, compounds **2, 4,** and epicatechin gallate **7** demonstrated solubility higher than 1,000 μg/mL with average logS_H2O_ and logS_pH7.4_ higher than (−2.51) and (−2.65), respectively.

**TABLE 3:** Solubility of compounds **1**–**7** as well as standard compounds in water and buffer solution with pH 7.4.

Compound	Mw	Sol. _(H2O)_ (μg/mL)	logS_H2O_	Sol. _(buffer 7.4)_ (μg/mL)	logS_pH 7.4_
1	216.28	33.33	−3.81	>160	−3.13
2	376.28	>1,000	>−2.58	298.6	−3.10
3	344.37	<6	<−4.76	<6	<−4.76
4	158.16	>1,000	>−2.20	>1,000	>−2.20
5	363.20	>160	>−3.36	>160	>−3.36
6	240.30	<6	<−4.60	<6	<−4.60
7	422.37	789.1	−2.75	>1,000	>−2.65
Rapamycin	914.17	<6	<−5.18	<6	<−5.18
Torkinib	308.34	<6	<−4.71	<6	<−4.71

### Biological properties

#### Cytotoxic effects of novel compounds examined *in vitro* in human normal and cancerous cells

**FIGURE 4:**
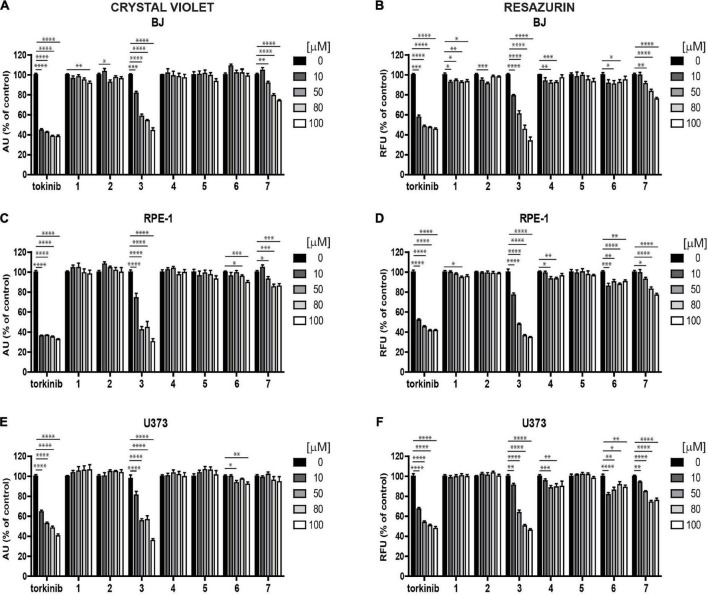
Screening of new compounds on proliferation of normal and cancerous cells. Normal human dermal fibroblasts BJ **(A,B)**, human immortalized retinal pigment epithelium RPE-1 **(C,D)**, and human glioblastoma U373 **(E,F)** cells were exposed to compounds **1**–**7** in the concentration range of 0–100 μM for 24 h and assayed by crystal violet **(A,C,E)** and resazurin **(B,D,F)** assays. Torkinib served as a reference compound. The experiment was done in triplicate. Data were normalized to untreated cells and plotted as mean ± SEM. Two-tailed Student’s *t*-test, ^****^p < 0.0001; ^***^p < 0.001; ^**^p < 0.01; *p < 0.05.

To determine the effect of novel compounds on cell proliferation, normal human skin fibroblasts (BJ), non-transformed telomerase-immortalized retinal pigment epithelial cells (RPE-1), and cancerous human glioblastoma cell line U373 were exposed to different concentrations of compounds **1–7** (0, 10, 50, 80, and 100 μM) for 24 h using torkinib as a reference compound. We have decided to use only torkinib as a representative of small-molecule mTOR inhibitors for all biological experiments. Pilot tests employing crystal violet determining the residual number of attached cells (Figures 4A,C,E) and resazurin assays measuring the metabolism of cell population (Figures 4B,D,F) revealed that only compound **3** exerted a significant suppressive effect on the readout of both assays with comparable potency to torkinib in all cell types tested. Other compounds (**1, 2, 4, 6**, and **7**) showed mild (although sometimes statistically significant) suppressive effect in dependence on the cell type used. Compound **5** showed no effect.

Next, we followed the cells exposed to compounds **1–7** and torkinib by time-lapse microscopy for 72 h (with a 1-h interval between captures) using the Incucyte SX1 platform. In concert with crystal violet and resazurin assays, only compound **3** (0, 10, 50, 80, and 100 μM) showed the effect on RPE-1 cell proliferation (Supplementary Figure 2A). Compound **7** (epicatechin gallate) showed a cytostatic effect at higher concentrations (80–100 μM). Similar effect was observed for U373 cells (Supplementary Figure 2B). Again, compound **7** showed cytostatic effect at higher concentration (100 μM).

**FIGURE 5:**
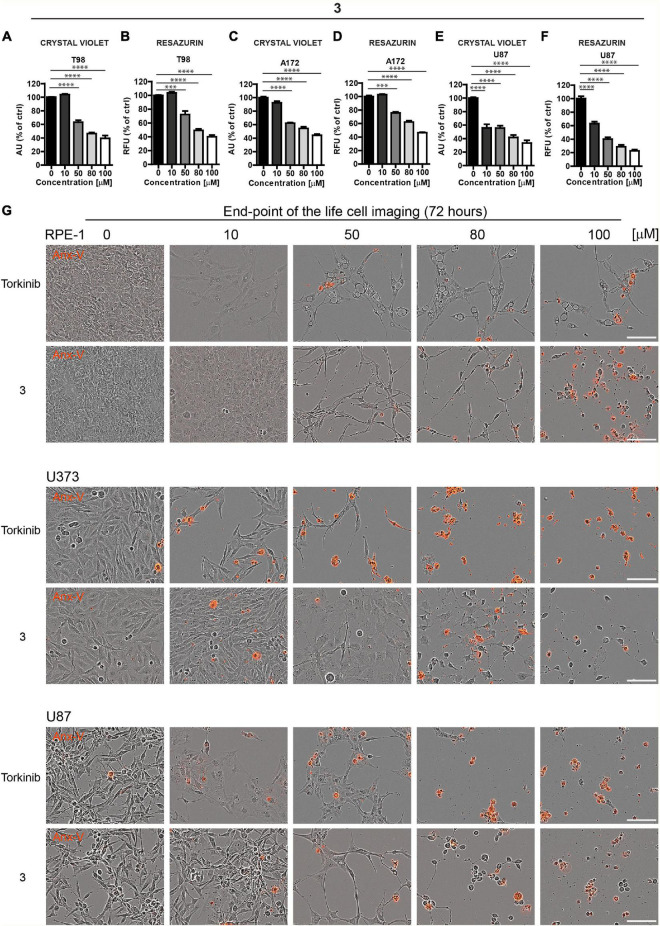
The cytotoxic effect of new compound **3** on glioblastoma cells. Human glioblastoma T98 **(A,B)**, A172 **(C,D)**, and U87 **(E,F)** cells were exposed to compound **3** in the concentration range of 0–100 μM for 24 h and assayed by crystal violet **(A,C,E)** and resazurin **(B,D,F)** assays. The experiment was done in duplicate. Data were normalized to untreated cells and plotted as mean ± SEM. Two-tailed Student’s *t*-test, ^****^p < 0.0001; ^***^p < 0.001. **(G)** Cytotoxic effect of torkinib and compound **3** (concentration range of 0–100 μM) demonstrated by time-lapse microscopy (72 h) using Incucyte SX1 platform. End-point images (72 h) are presented. Red color is annexin V staining. Bar, 100 μm.

Based on these screens, we broadened the tests of the most effective compound **3** using other glioblastoma cell lines, T98, A172, and U87. Like U373 cells, all tested glioblastoma cells, including T98 cell line characterized by higher cell death resistance compared to other glioblastoma cell lines, were significantly affected by compound **3** (see Figures 5A–F for crystal violet and resazurin assays). Further, in proliferating U87 cells, time-lapse microscopy showed that compound **3** (100 μM) caused rapid loss of cell spreading with a lack of mitotic activity and progressively occurring cell death. A similar effect was observed for torkinib (100 μM; Supplementary Figure 2C).

The induction of apoptosis by compound **3** was confirmed by annexin V staining (Figure 5G).

To conclude, compound **3,** compared to the rest of the novel series of substances, showed a significant cytotoxic effect on normal and cancerous cells, including a series of glioblastoma cell lines with similar potency to reference compound torkinib.

#### Senolytic effects of novel compounds determined *in vitro* in human normal and cancerous cells

**FIGURE 6:**
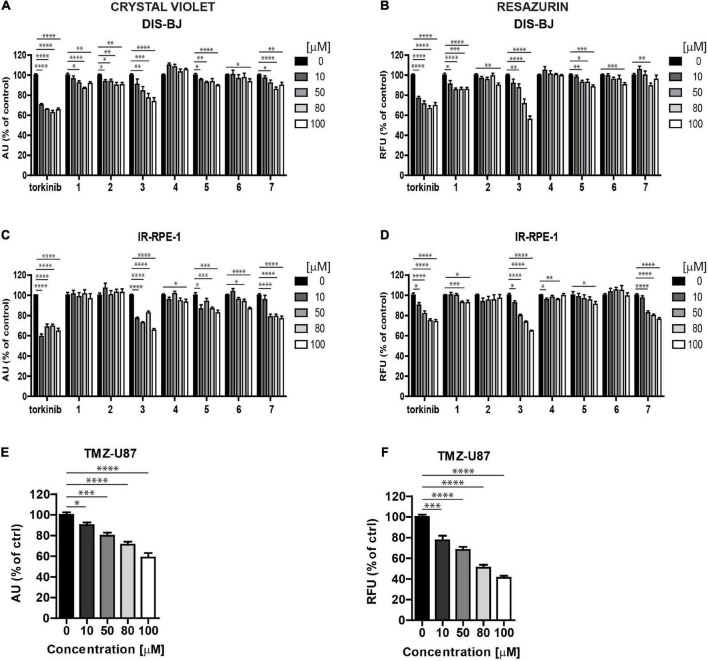
Screening of new compounds on senescent cells. Ionizing radiation (IR)-induced RPE-1 **(A,B)** and docetaxel (DTX)-induced BJ **(C,D)** cells were exposed to compounds **1**–**7** in the concentration range of 0–100 μM for 24 h. Torkinib served as a reference compound. Temozolomide (TMZ)-induced glioblastoma U87 cells **(E,F)** were exposed to compound **3** in the concentration range of 0–100 μM for 24 h. The senescent cell response was detected by crystal violet **(A,C,E)** and resazurin **(B,D,F)** assays. The experiment was performed in triplicate. Data were normalized to untreated cells and plotted as mean ± SEM. Two-tailed Student’s *t*-test, ^****^p < 0.0001; ^***^p < 0.001; ^**^p < 0.01; *p < 0.05.

Senescent cells are, in general, more resistant to apoptosis, which can be restored by mTOR inhibition (Laberge et al., 2015). To examine the cytotoxicity of novel compounds against non-transformed senescent cells (i.e., senolytic effect), ionizing radiation (IR)-induced and drug-induced modes of cellular senescence in RPE-1 and BJ cells, respectively, were employed (see “Materials and methods” for details). The development of senescence was confirmed by routinely used assays (an increase of senescence-associated beta-galactosidase activity, expression of cyclin-dependent kinase inhibitor p21waf1, and halt of DNA replication by EdU-incorporation assay; see Supplementary Figures 3A–G). Both IR-induced senescent RPE-1 and docetaxel (DTX)-induced senescent BJ cells were significantly sensitive to compound **3** (Figures 6A–D), however, in a non-selective manner (i.e., they were less sensitive to compound **3** than proliferating counterparts) as detected by crystal violet and resazurin assays (Supplementary Figures 4A,B). In addition, a mild but selective effect on crystal violet readout of γ-irradiated RPE-1 cells was observed for compound **5** (Supplementary Figure 4B). Reference compound torkinib exerted the non-selective effect in crystal violet and resazurin assays.

**FIGURE 7:**
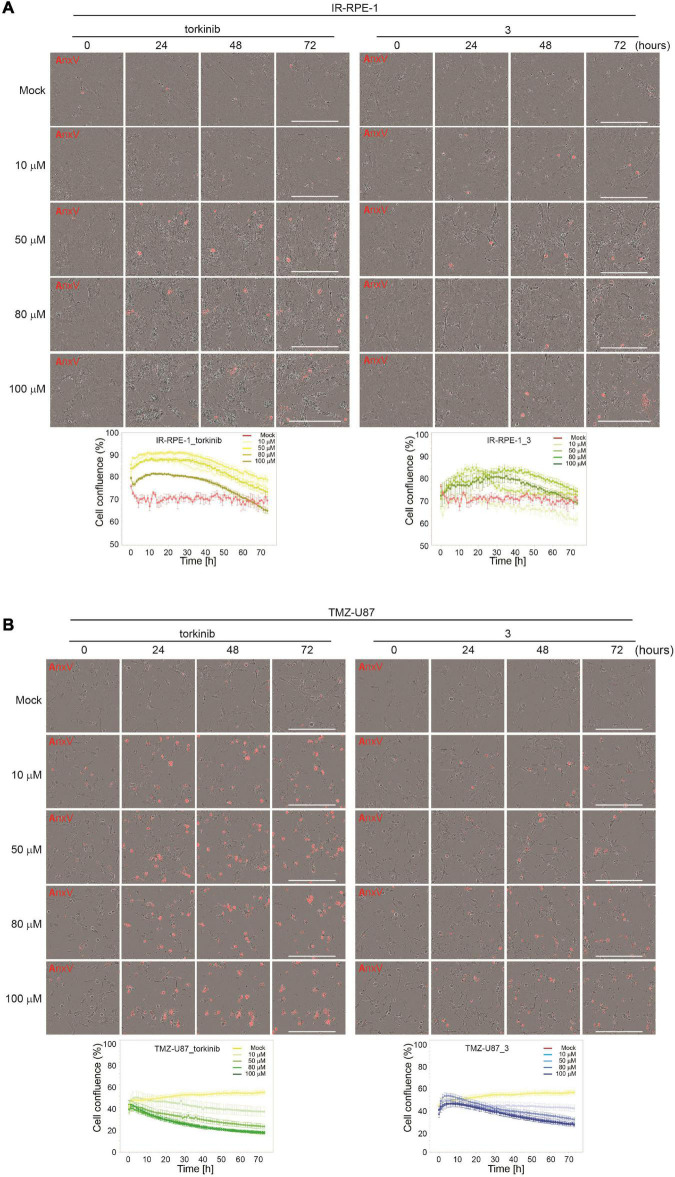
Time-lapse microscopy of torkinib and compound **3** using Incucyte SX1 platform. γ-irradiated RPE-1 **(A)** and TMZ-induced U87 **(B)** senescent cells were exposed to torkinib and compound **3** in a concentration range of 0–100 μM for 72 h. Images were acquired every 60 min. Note that the first image was acquired 30 min (corresponding to 0 h in graph plots) after adding compounds. The graphs plot changes in cell confluence during the treatment. Average cell confluence and standard error from four images are shown. End-point images (72 h) are presented. Red color is annexin V staining. Bar, 400 μm.

To evaluate the effect of compound **3** on the senescent cell phenotype of non-transformed cells, time-lapse microscopy of IR-senescent RPE-1 cells exposed to compound **3** (100 μM) and torkinib (100 μM) was performed for 72 h. Torkinib caused in IR-RPE-1 senescent cells rapid onset of vacuolization (after approximately 1 h of exposure) with partial loss of adhesion (decreased cells covered area) but only with a few senescent cells dying (Figure 7A). Compound **3** generated a similar phenotypic manifestation in IR-RPE-1 senescent cells as torkinib except for vacuolization. These findings indicate that both compounds induced a rather senomorphic than senolytic effect in IR-RPE-1 cells.

As cancer cells can undergo a senescent-like phenotype when exposed to subapoptotic doses of chemotherapeutics, we tested the response of compound **3** on glioblastoma cell line U87 induced to senescence by chemotherapeutics temozolomide (TMZ, 100 μM) used in the therapy of glioblastoma in clinics (Karachi et al., 2018). As shown in Figures 6E,F, compound **3** affected the readout of crystal violet and resazurin assays similarly to proliferating U87 cells. Moreover, time-lapse microscopy revealed that compound **3** induced the loss of cell adhesion followed by cell death as detected by annexin V staining (Figure 7B). Note that torkinib exerted the same effect on TMZ-U87 cells.

Together, compound **3** and reference compound torkinib affected the senescent phenotype of non-transformed cells, apparently without the induction of extensive cell death. Contrarily, both compounds induced massive cell death of temozolomide-induced glioblastoma senescent-like cells. This type of selectivity between normal and cancerous senescent cells can be desirable in adjuvant therapy regimes of cancer-related senescence induced by radiochemotherapy, as only senescent cells originating from cancer but not normal cells will be removed.

#### Screening for inhibition of mechanistic target of rapamycin kinase activity by novel compounds

**FIGURE 8:**
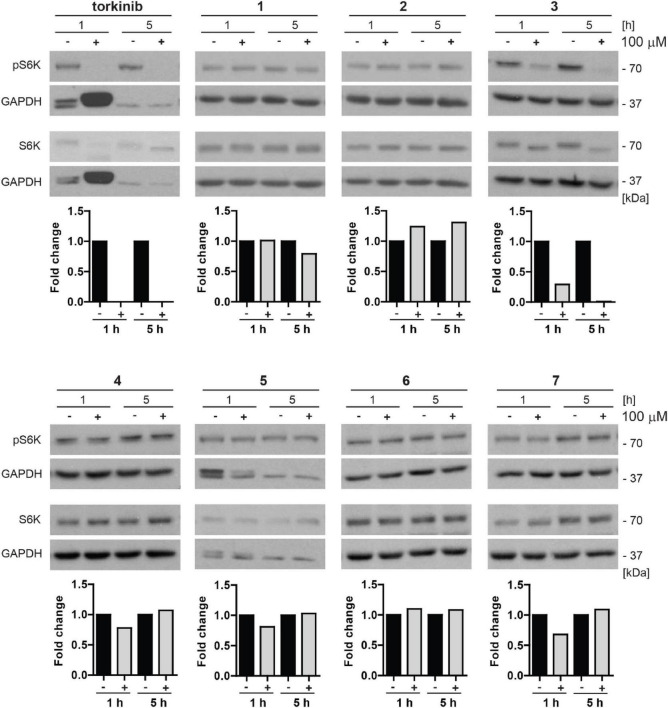
Inhibitory effect of new compounds on mTORC1 kinase substrate p70 S6 kinase in BJ cells. All compounds (**1**–**7**) and control compound torkinib were tested at a concentration of 100 μM for 1- and 5-h-long exposure. Whole-cell lysates were subjected to SDS-PAGE/immunoblotting analysis and probed for phospho-p70 S6K (Thr389) and total p70 S6K. GAPDH was used as a loading control. Note that the robustly elevated level of GAPDH after 1 h exposure to torkinib observed in all experimental replicates is not a loading artifact. Quantitative analysis of immunoblots was done by Image J 1.48v program with GAPDH as the internal control.

To examine whether novel compounds could inhibit mTOR kinase, BJ cells were exposed to novel compounds **1**–**7** (100 μM) for 1 and 5 h, and the activity of mTORC1 was detected by determining specific phosphorylation levels (threonine 389) of its substrate p70 S6 kinase (S6K), by immunoblotting with specific antibodies. As shown in Figure 8, the inhibitory effect on Thr389 S6K phosphorylation was detected only for compound **3** and torkinib. The rest of the drugs showed mild or no effect on Thr389 S6K phosphorylation. Note that the suppression of S6K phosphorylation was associated with increased electrophoretic protein mobility of S6K protein.

Thus, in correlation with the cytotoxic effect, compound **3** inhibits phosphorylation of mTORC1 substrate p70 S6 kinase indicating its inhibitory effect on mTORC1 kinase.

#### Compound 3 is a direct mechanistic target of rapamycin complex 1 kinase inhibitor with activity in various normal and cancer cells

**FIGURE 9:**
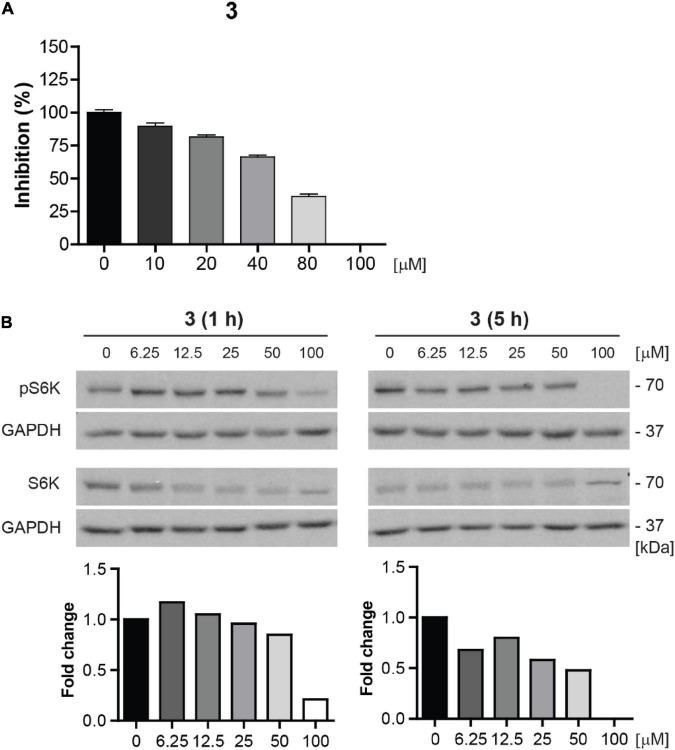
Compound **3** is the direct inhibitor of mTORC1 kinase. **(A)** *In vitro* mTOR kinase enzyme assay of compound **3** tested in the concentration range of 0–100 μM. The percentage of mTOR kinase activity inhibition is plotted. **(B)** Inhibition of mTORC1 kinase activity assayed in BJ cells after 1- and 5-h-long exposure to compound **3** (0–100 μM) by determination of phosphorylation level of mTORC1 substrate p70 S6K with anti-Thr389 p70 S6K and total p70 S6K antibodies. GAPDH was used as a loading control. Quantitative analysis of immunoblots was done by Image J 1.48v program with GAPDH as the internal control.

Due to the highest cytotoxicity and inhibitory effect on mTORC1 kinase activity, compound **3** was selected for further testing. To corroborate whether compound **3** is a direct inhibitor of mTOR kinase, we performed *in vitro* mTOR kinase enzyme assay (see “Materials and methods” section). As shown in Figure 9A, compound **3** inhibited mTOR kinase activity from the concentration of 10 μM with complete inhibition at 100 μM with IC_50_ value approximately 70 μM.

To further validate the inhibition of mTOR activity by compound **3**, we examined the inhibitory effect of compound **3** on S6K phosphorylation in the concentration range 0–100 μM in proliferating BJ cells by immunoblotting. The apparent effect of compound **3** on inhibition of S6K phosphorylation was revealed from the concentration 6.25 μM after 5 h-long exposure (Figure 9B).

**FIGURE 10:**
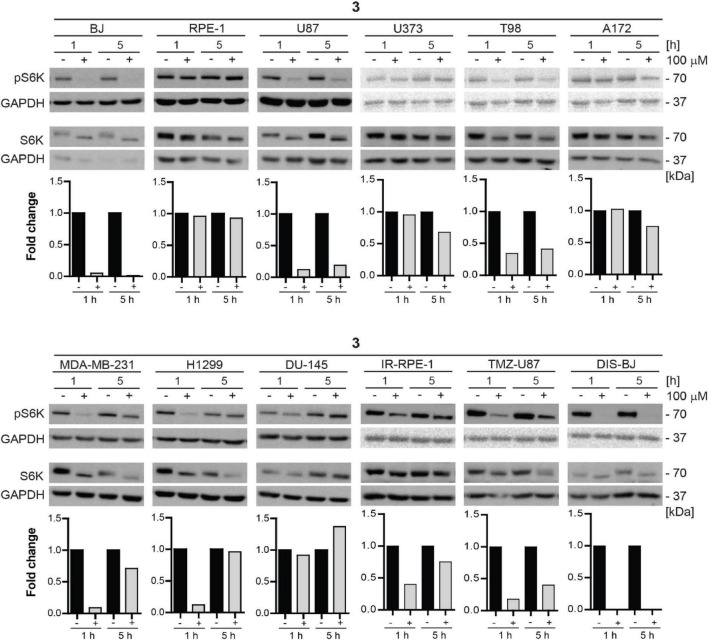
Inhibitory effect of new compound **3** on mTORC1 kinase and its substrate S6 kinase in various cell lines. Human dermal fibroblasts BJ, immortalized retinal pigment epithelium RPE-1, glioblastoma U87, U373, T98, A172, breast adenocarcinoma MDA-MB-231, lung cancer H1299, prostate cancer DU-145 cells, ionizing radiation (IR)-induced RPE-1, docetaxel (DTX)-induced BJ, and temozolomide (TMZ)-induced U87 were exposed to compound **3** (100 μM) for 1- and 5-h-long exposure. Whole-cell lysates were subjected to SDS-PAGE/immunoblotting analysis and probed for phospho-p70 S6K (Thr389) and total p70 S6K. GAPDH was used as a loading control. Quantitative analysis of immunoblots was done by Image J 1.48v program with GAPDH as the internal control.

Additionally, we tested the inhibitory effect of compound **3** on S6K phosphorylation in a series of human non-transformed and cancer-derived cell lines. For this purpose, RPE-1, glioblastoma U87, U373, T98, and A172, breast adenocarcinoma MDA-MB-231, lung cancer H1299, and prostate cancer DU-145 cell lines were exposed to compound **3** (100 μM) for 1 and 5 h and the level of mTORC1 substrate phospho-p70 S6K was assayed by immunoblotting. Notably, as shown in Figure 10, the most potent inhibitory effect of compound **3** was detected in glioblastoma U87 cells after 1 h-long exposure maintaining a reasonable level also after 5 h. Marked but transient inhibition of Thr389 S6K phosphorylation was also observed in T98, MDA-MB-231, and H1299 cells, whereas no effect was detected in RPE-1 and DU-145 cells, indicating cell type-dependent (in)sensitivity to mTORC1 inhibition by compound **3**.

Furthermore, we tested the mTORC1 inhibitory activity of compound **3** in senescent cells. For this purpose, docetaxel-induced BJ, ionizing radiation-induced RPE-1, and TMZ-induced U87 senescent cells were exposed to compound **3** (100 μM) for 1 and 5 h, and the level of mTORC1 substrate phospho-p70 S6K was assayed by immunoblotting as above. The robust effect on p70 S6K phosphorylation was detected in docetaxel-induced BJ cells, whereas a less pronounced effect was revealed in temozolomide-induced U87 and ionizing radiation-induced RPE-1 cell lines.

In conclusion, compound **3** inhibitory effect on mTORC1 activity is cell type-dependent with inhibitory activity exerted in glioblastoma, breast, and lung cancer cell lines. From a series of tested tumor cells, the absence of inhibitory activity was observed only in prostate cancer cell line DU-145. Inhibitory activity of compound **3** in senescent cells was comparable to proliferating cells, only the impact on inhibition of mTORC1 activity in IR-induced senescent RPE-1 cells was more apparent than in proliferating cells.

#### Detection of mechanistic target of rapamycin complex 2 kinase inhibition activity of compound 3

It is generally known that only mTORC1 is acutely sensitive to rapamycin, whereas mTORC2 is inhibited just after chronic administration of the mentioned drug. More recently, it has been revealed that particularly reduced activity of mTROC2 is responsible for the adverse effects of rapamycin treatment, such as glucose intolerance or hepatic insulin resistance (Lamming, 2014). On the other hand, dual inhibition of both mTORC1 and mTORC2 complexes is proposed as a more effective treatment of cancer cells.

**FIGURE 11:**
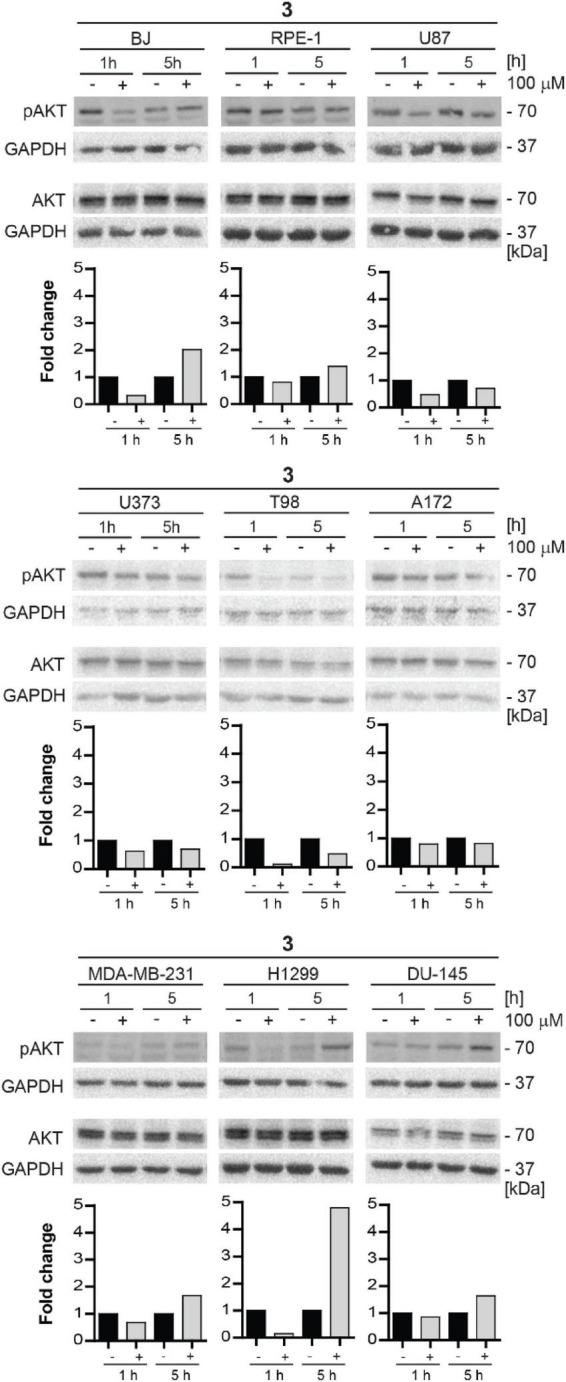
Inhibitory effect of new compound **3** on mTORC2 kinase and its substrate AKT kinase in various cell lines. Human fibroblasts BJ, immortalized retinal pigment epithelium RPE-1, glioblastoma U87, U373, T98, and A172, breast adenocarcinoma MDA-MB-231, lung cancer H1299, and prostate cancer DU-145 cells were exposed to compound **3** (100 μM) for 1 and 5 h. Whole-cell lysates were subjected to SDS-PAGE/immunoblotting and probed for phospho-AKT (Ser473) and total AKT with specific antibodies. GAPDH was used as a loading control. Quantitative analysis of immunoblots was done by Image J 1.48v program with GAPDH as the internal control.

For this reason, we decided to test the inhibitory effect of compound **3** on phosphorylation of mTORC2 substrate AKT using several human normal, immortalized, and cancer-derived cell lines. BJ, RPE-1, U87, U373, T98, A172, MDA-MB-231, H1299, and DU-145 cells were exposed to compound **3** (100 μM) for 1 and 5 h and the level of AKT serine 473 (Ser473) phosphorylation was assayed by immunoblotting. Transient inhibition (after 1 h) of Ser473 AKT phosphorylation was observed in BJ, RPE-1, U87, U373, T98, A172, MDA-MB-231, H1299, and DU-145 cells. However, in several cell types (BJ, RPE-1, MDA-MB-231, H1299, and DU-145), the AKT phosphorylation was higher after 5 h of exposure than in control cells (Figure 11). Notably, the most potent inhibitory effect of compound **3** was revealed in glioblastoma T98 cells after 1 h, maintaining inhibitory level also after 5 h of exposure. Overall, compound **3** harbors the transient inhibitory effect on mTORC2 activity in the cell type-dependent manner.

#### Determination of anti-senescence-associated secretory phenotype activity of compound 3

Progressive accumulation of senescent cells can deplete the organism of functional cells required for tissue repair and regeneration, such as stem and progenitor cells. Therefore, it seems logical to remove senescent cells to solve the problem. However, the studies in different murine models showed defects in wound healing when senescent cells were eliminated (Baker et al., 2011; Demaria et al., 2014). Thus, compounds modulating the senescent secretome seem to be the better alternative, since the production of factors secreted by senescent cells termed senescence-associated secretory phenotype (SASP) is considered a primary mediator of the detrimental effects of senescent cells (Coppé et al., 2008; Wang et al., 2017). Chronic, low-grade inflammation, characterized by elevated levels of circulating cytokines and increased immune infiltration associated with inflammaging, fuels loss of resilience, and increases the risk of age-related diseases (Franceschi and Campisi, 2014; Birch and Gil, 2020).

**FIGURE 12:**
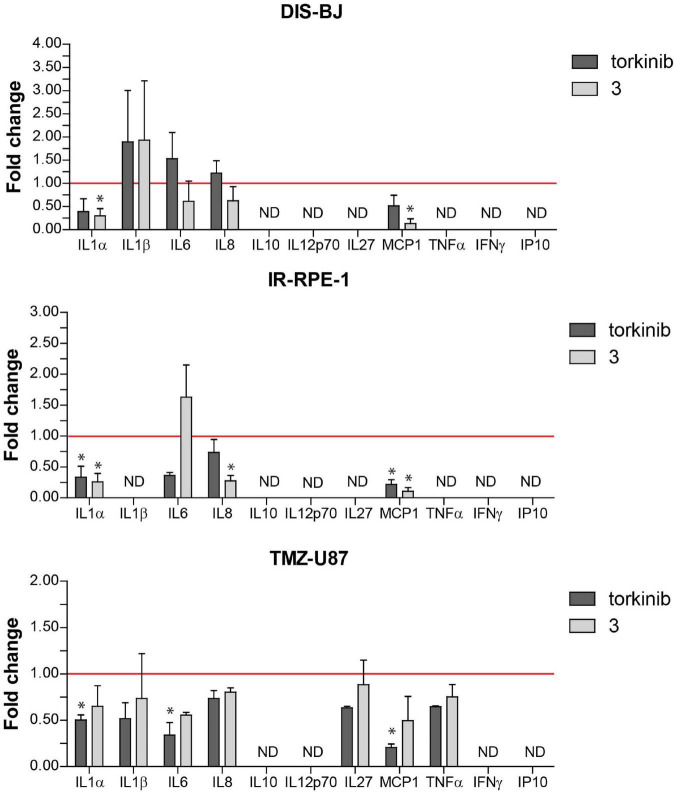
Secretion of pro-inflammatory cytokines by senescent cells after exposure to compound **3** and torkinib. Determination of 11 pro-inflammatory cytokines IL-1α, IL-1β, IL-6, IL-8, IL-10, IL-12p70, IL-27, MCP-1, IFNγ, TNFα, and IP-10, and IP-10 in docetaxel-(DIS-BJ), ionizing-radiation- (IR-RPE-1), and temozolomide-induced (TMZ-U87) senescent cells exposed to compound **3** and torkinib (50 μM) for 24 h. Conditioned media were collected 24 h after treatment and analyzed for the level of cytokines by FACS analysis. The experiment was done in triplicate (TMZ-U87 in duplicate). Data were normalized to untreated cells as fold induction to control (set as 1, red line) and plotted as mean ± SEM. ND, not detected. Two-tailed Student’s *t*-test, *p < 0.05.

To evaluate the effect of compound **3** on a pro-inflammatory component of SASP, we measured the level of 11 pro-inflammatory cytokines IL-1α, IL-1β, IL-6, IL-8, IL-10, IL-12p70, IL-27, MCP-1, IFNγ, TNFα, and IP-10 in culture media conditioned by three models of senescent cells (DIS-BJ, IR-RPE-1, and TMZ-U87) before and after 24 h-long incubation of senescent cells with compound **3** and torkinib (50 μM). As shown in Figure 12, the impact of both drugs on the level of inflammatory cytokines was drug- and cell type-dependent. Importantly, compound **3** decreased IL-1α, IL-8, and MCP-1 consistently in all tested senescence models. The effect of compound **3** on IL-6 was variable. The apparent decrease of IL-6 was observed in DIS-BJ and TMZ-U87 senescent cells. Notably, both compound **3** and torkinib suppressed all detectable cytokines (i.e., IL-1α, IL-1β, IL-6, IL-8, IL-27, MCP-1, and TNFα) in TMZ-induced U87 senescent cells.

#### *In vivo* tolerability

**FIGURE 13:**
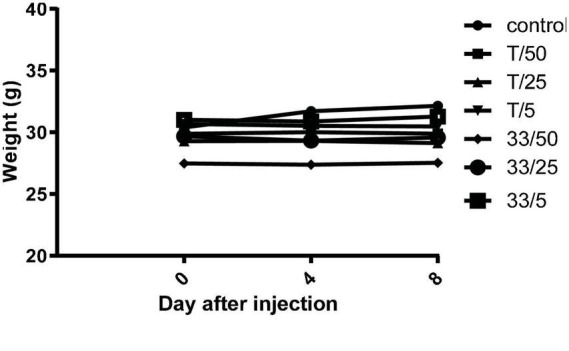
Mice weights after repeated administration of increasing doses (5, 25, and 50 mg/kg body weight) of compound **3** (33) and torkinib (T).

To investigate *in vivo* tolerability of new substances, compound **3** and torkinib were repeatedly (three times) perorally administered at increasing doses (5, 25, and 50 mg/kg body weight), three times on days 0, 3, and 5. Indeed, no apparent signs of the whole body or organ toxicities and no changes in mice behavior, as well as changes in the body weights were observed after the treatments (Figure 13).

Further, the blood count analysis revealed that although there were changes between samples, no abnormal changes were detected after administration of tested compounds (**3** and torkinib). The data are presented in Table 4.

**TABLE 4:** Blood counts of mice treated with compound **3** (33) and torkinib (T).

		WBC	Neu	l_vm_	Mon	Eos	RBC	HGB	HOT	MCV	MCH	MCHC	RDW-CV	RDW-SD	PLT	MPV	PDW	PCT
Control	AVG	11.60	1.94	8.96	0.40	0.52	9.92	156.00	0.54	54.05	15.75	290.50	0.14	31.95	491.00	5.45	14.85	2.68
	SD	12.05	0.74	1.32	0.30	0.03	0.15	11.31	0.02	1.20	0.92	10.61	0.01	2.05	108.89	0.21	0.07	0.49
T/50	AVG	8.70	1.25	6.32	1.09	0.04	9.95	153.67	0.53	53.27	15.50	290.33	0.14	31.07	873.67	5.43	14.67	4.73
	SD	0.99	0.21	1.55	0.58	0.04	0.93	8.50	0.03	2.39	0.61	1.53	0.01	3.72	164.61	0.25	0.15	0.73
T/25	AVG	8.14	0.79	6.68	0.59	0.08	9.96	154.00	0.52	51.90	15.50	298.00	0.14	29.23	712.00	5.30	14.73	3.79
	SD	2.61	0.26	2.08	0.25	0.04	0.78	10.39	0.05	0.56	0.17	5.57	0.00	0.49	425.39	<0.01	0.15	2.28
T/5	AVG	10.43	0.99	8.67	0.73	0.04	9.25	146.00	0.49	53.73	15.97	296.67	0.14	31.77	690.67	5.33	14.57	3.59
	SD	3.13	0.31	2.50	0.31	0.03	1.68	15.87	0.05	4.91	1.33	2.52	0.01	5.40	369.88	0.32	0.12	1.79
33/50	AVG	8.25	0.86	6.23	0.72	0.43	9.99	150.67	0.52	52.07	15.10	289.33	0.13	29.03	751.67	5.20	14.70	3.92
	SD	2.88	0.45	1.95	0.28	0.26	0.40	7.09	0.02	0.25	0.17	3.51	0.01	2.34	86.01	0.20	0.17	0.42
33/25	AVG	11.49	1.47	8.71	0.52	0.74	8.76	136.00	0.46	52.77	15.53	294.00	0.15	32.23	330.00	5.37	14.93	1.77
	SD	4.82	0.88	4.09	0.17	0.58	1.74	26.96	0.09	0.75	0.15	7.55	<0.01	0.70	147.89	0.15	0.25	0.74
33/5	AVG	12.56	1.65	9.60	0.39	0.88	8.28	131.00	0.44	53.70	15.90	296.33	0.14	31.27	677.33	5.67	15.20	3.88
	SD	4.63	0.71	3.86	0.16	0.13	1.25	13.00	0.04	2.95	0.95	2.08	0.01	2.25	163.16	0.64	0.61	1.32

Spleen cells were analyzed by flow cytometry for possible changes in the percentages of important selected immune cell populations (CD45^+^, CD4^+^, CD8^+^, and CD11b^+^/Gr-1^+^; Table 5). No significant differences were found between samples from the control and treated groups.

**TABLE 5:** Selected spleen cell populations of mice treated with different doses of compound **3** and torkinib (T).

	CD45^+^	CD4^+^	CD8^+^	Cd11b^+^/Gr-1^+^
Control	86.2	14.9	9.6	1.4
T/5	92.0	15.5	10.4	0.9
T/25	96.7	15.4	9.8	0.6
T/50	84.0	16.6	10.2	1.3
**3**/5	94.3	19.0	11.4	0.7
**3**/25	95.9	15.9	9.6	1.0
**3**/50	97.2	16.0	9.8	0.8

CD4^+^, CD8^+^, and CD11b^+^/Gr-1^+^ are presented as the percentage from the CD45^+^ cells.

#### Histopathological analysis

Histopathological analysis of the spleen, kidney, and liver tissue samples revealed pathological changes in these organs compared to treated animals. In general, they were more pronounced in the samples from the torkinib-treated mice, as compared to the compound **3**-treated mice.

**FIGURE 14:**
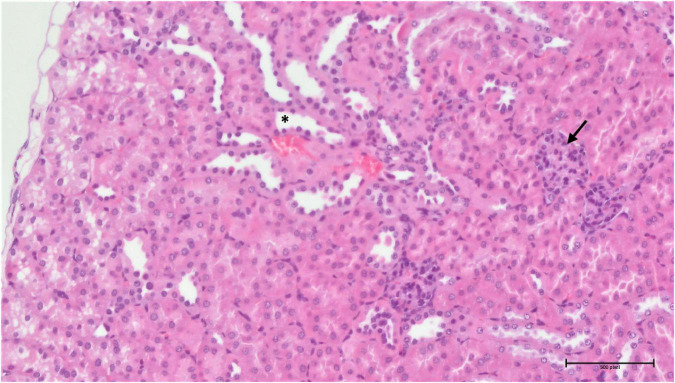
Group 3/25 – kidneys (HE, 200×): narrowing of Bowman’s space (arrow), focal tubular atrophy (asterisk).

**FIGURE 15:**
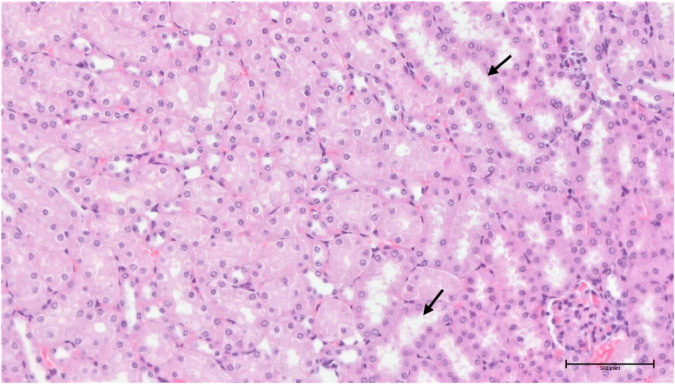
Group 3/50 – kidneys (HE, 200×): multifocal mild tubular damage (arrow).

Analysis of the compound **3** potential toxic effects demonstrated that the pathological manifestation in the spleen appeared to be almost identical in all three different doses, with varying degrees of extramedullary haematopoiesis (Supplementary Figure 5). However, the situation was completely different in the liver and kidneys. In the liver, there was apparent just minimal evidence of damage, although there were signs of incipient and discrete centrilobular-centrozonal necrosis of hepatocytes in a dose-dependent manner (Supplementary Figure 6). The most pronounced pathological changes were evident in the kidneys (cortex and medulla), at the glomerular and tubular levels – the latter being predominant (Figures 14, 15). Again, the parenchymal damage was directly proportional to the treatment dose.

**FIGURE 16:**
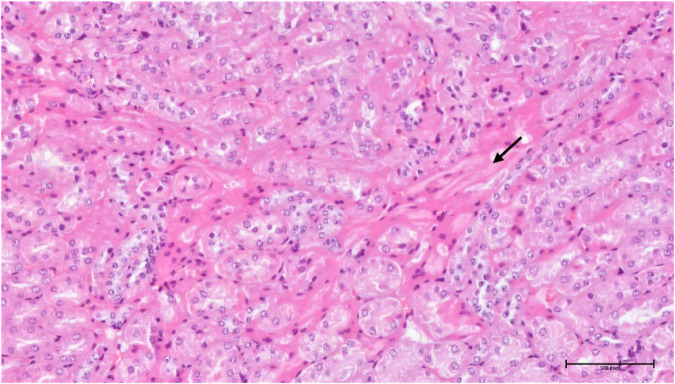
Group torkinib/5 – kidneys (HE, 200×): focal interstitial fibrosis (arrow), mild tubular damage, hyaline droplets.

In the samples from the torkinib-treated mice, the pathological changes were more pronounced, compared to those from the compound **3**-treated mice, with the exception of the spleen, in which the same minimal to moderate signs of extramedullary haematopoiesis were observed. Again, the most affected organ was the kidney in the glomeruli and the tubular system. Here, in addition to the kidneys of the substance **3**-treated mice, signs of fibrosis were already evident (Figure 16). The pathological changes were again proportional to the torkinib doses used for the treatments.

## Conclusion

For decades, one of the most debated questions in gerontology was whether aging is a disease or the norm. Indeed, aging is “the sum of all age-related diseases”. It is not so much a matter of determining whether aging is a disease or not, an important thing is that aging is treatable (Blagosklonny, 2018a). Currently, rapamycin has the strongest experimental support as a potential anti-aging therapeutic in mammals. Unlike many other compounds claimed to influence longevity, rapamycin has been repeatedly tested in long-lived, genetically heterogenous mice, in which it extends both mean and maximum lifespans. However, rapamycin’s inappropriate physicochemical properties and a growing list of side effects make it doubtful that rapamycin would ultimately be beneficial in humans (Lamming et al., 2013).

The text above carries two important messages, namely, that mTOR seems to be a suitable biological target for anti-aging research and that there is a need to discover new substances that could pharmacologically affect mTOR without the restrictions mentioned above. Rapamycin is an allosteric mTOR inhibitor. However, there is also a group of ATP-binding mTOR inhibitors (competitive inhibitors) that have gradually come to prominence. Originally, ATP-binding mTOR inhibitors were developed to treat cancer. However, more detailed research on these compounds pointed out a wide window between their gerosuppressive and cytotoxic effects since, at optimal gerosuppressive concentrations, ATP-binding mTOR inhibitors exert only a mild cytotoxic effect (Leontieva and Blagosklonny, 2016). One of such substances seems to be also our compound **3**.

Initially, it is worthy to notice that unlike standard compounds (torkinib and rapamycin), compound **3** demonstrated better physicochemical properties as well as the higher MPO CNS score, suggesting that this substance will be able to reach the “all-body compartments” equally. Such property is highly desirable since one of the key goals of ideal anti-aging therapy is to stop geroconversion within the whole organism, including CNS. Additionally, the *in silico* study confirmed that ligand **3** interacts with crucial residues (Trp2239 and Val2240) of mTOR kinase. A series of cytotoxicity experiments revealed that highlighted compound **3** exerts the cytotoxic effect not only in proliferating but also in senescent normal and cancerous cells. Such finding just confirms possible multiple applications of ATP-binding mTOR inhibitors, i.e., to slow down the aging process as well as to treat cancer in a dose-dependent manner. Regarding selectivity toward mTORC1 and mTORC2, compound **3** again excelled in inhibiting mTORC1 in normal and cancerous cells, while in case of mTORC2, it proved its inhibitory potential preferentially in cancerous cell lines. Although the IC_50_ value of **3** (70 μM) does not reaches the IC_50_ value of torkinib (8 nM) (Oleksak et al., 2022), it is still the task of further research whether the strongest inhibition of mTOR is needed to induce a gero-suppressive effect or, on the contrary, whether only a modulatory effect will be sufficient to slow aging and concurrently to prevent the development of adverse effects. The promising anti-aging drug should also affect the production of factors secreted by senescent cells, termed SASP, since these factors are considered to be the primary mediators of harmful effects of senescent cells leading finally to increased risk of age-related diseases. Lead compound **3** consistently decreased IL-1α, IL-8, and MCP-1 levels in all tested senescence models. In light of outstanding *in vitro* results, the final comment is deserved to *in vivo* tolerability and toxicity. Even at the concentration of 50 mg/kg body weight, administered perorally, the animals did not exert any symptoms of intoxication. Detailed histopathological analysis revealed several pathological changes in the kidney, however, such changes were less pronounced than in torkinib-treated samples.

In conclusion, this work clearly demonstrates the therapeutic potential of ATP-binding mTOR inhibitors not only in cancer research, but also in longevity studies. However, to obtain the proof-of-concept for outstanding results of compound **3**, advanced *in vivo* effectiveness experiments on aged animals are required.

## Data availability statement

The original contributions presented in the study are included in the article/Supplementary material, further inquiries can be directed to the corresponding authors.

## Ethics statement

The animal study was reviewed and approved by Institutional Animal Care Committee of the Institute of Molecular Genetics, Prague.

## Author contributions

DS and MB: *in silico* study. ZC and PO: chemical synthesis. RA and AS: physico-chemical analysis. DR, PV, and JN: *in vitro* experiments. MR, RM, and ON: *in vivo* experiments. KK and MV: sources. EN and ZH: manuscript writing. All authors contributed to the article and approved the submitted version.
