# Supplementary material for: Discovery of small molecule mechanistic target of rapamycin inhibitors as anti-aging and anti-cancer therapeutics
Source: Front Aging Neurosci. 2022 Dec 6;14:1048260. doi: 10.3389/fnagi.2022.1048260 (PMC9767416; doi:10.3389/fnagi.2022.1048260)
Supplement: Supplementary file 1 [file Data_Sheet_1.pdf]

Supplementary Material

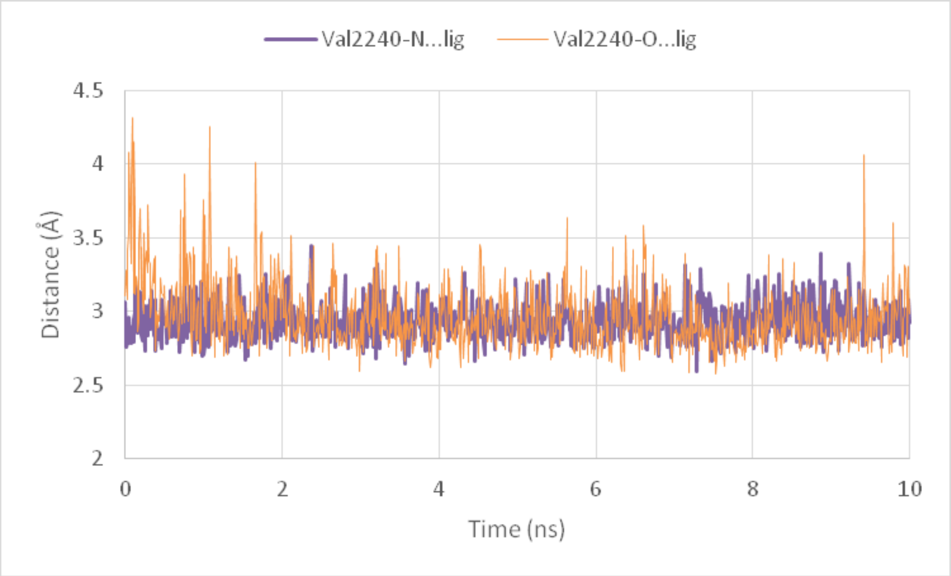

Figure S1. Distance changes between residues forming key interactions and ligand **3**

A

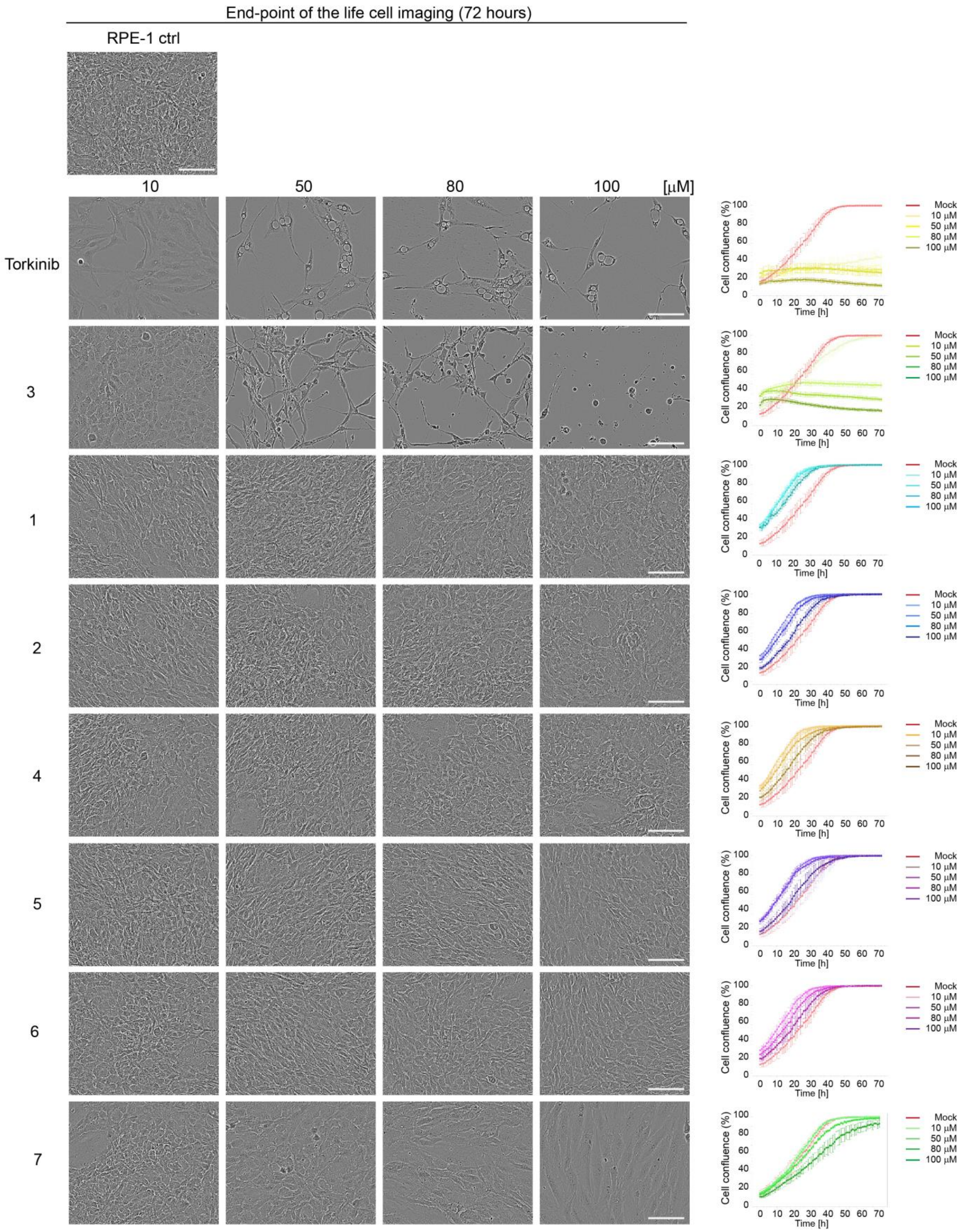
End-point of the life cell imaging (72 hours)
RPE-1 ctrl
10
50
80
100
[μM]
Torkinib
3
1
2
4
5
6
7
Cell confluence (%)
Time [h]
Mock
10 μM
50 μM
80 μM
100 μM

B

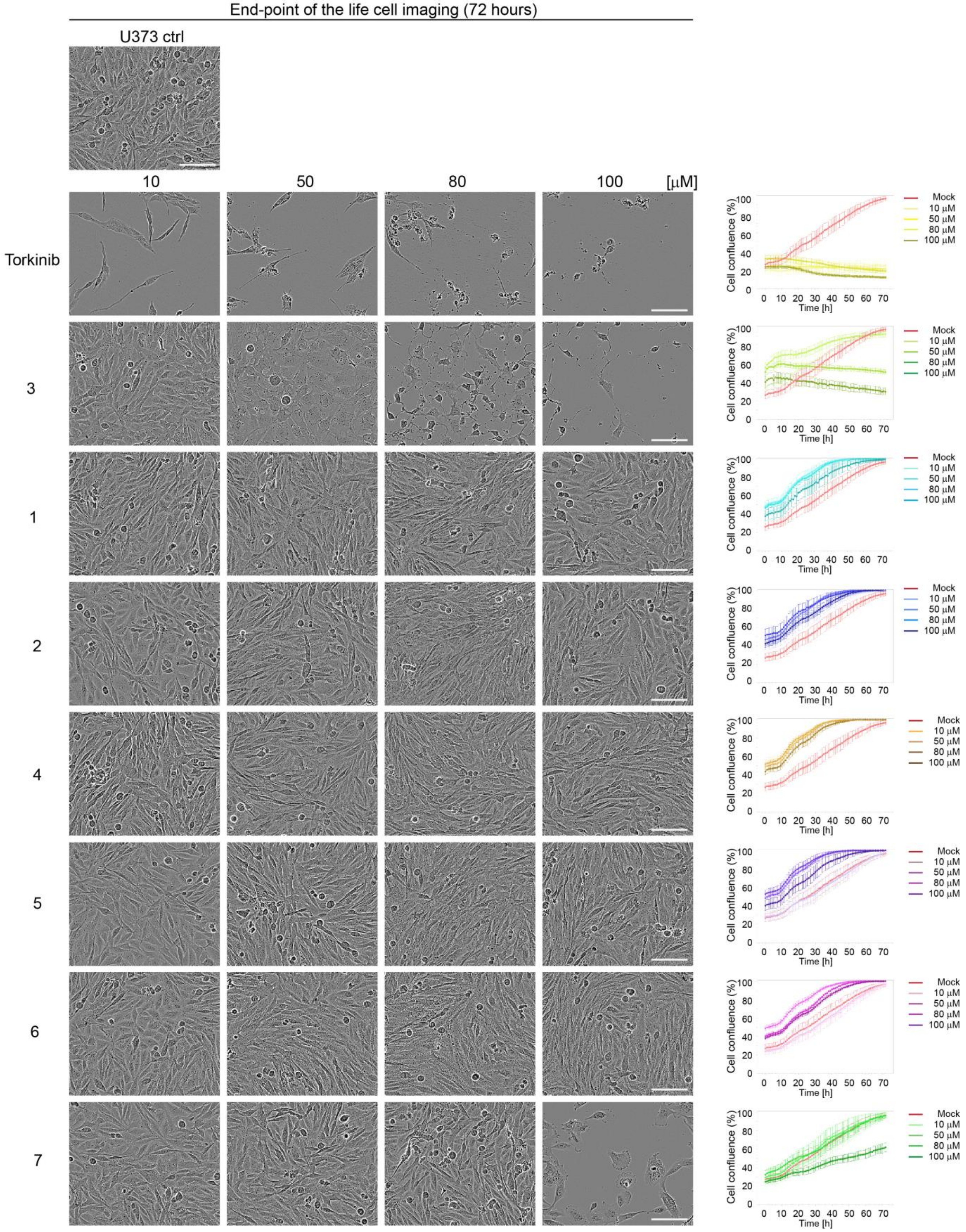

End-point of the life cell imaging (72 hours)
U373 ctrl
10
50
80
100
[μM]
Torkinib
3
1
2
4
5
6
7
Cell confluence (%)
Time [h]
Mock
10 μM
50 μM
80 μM
100 μM

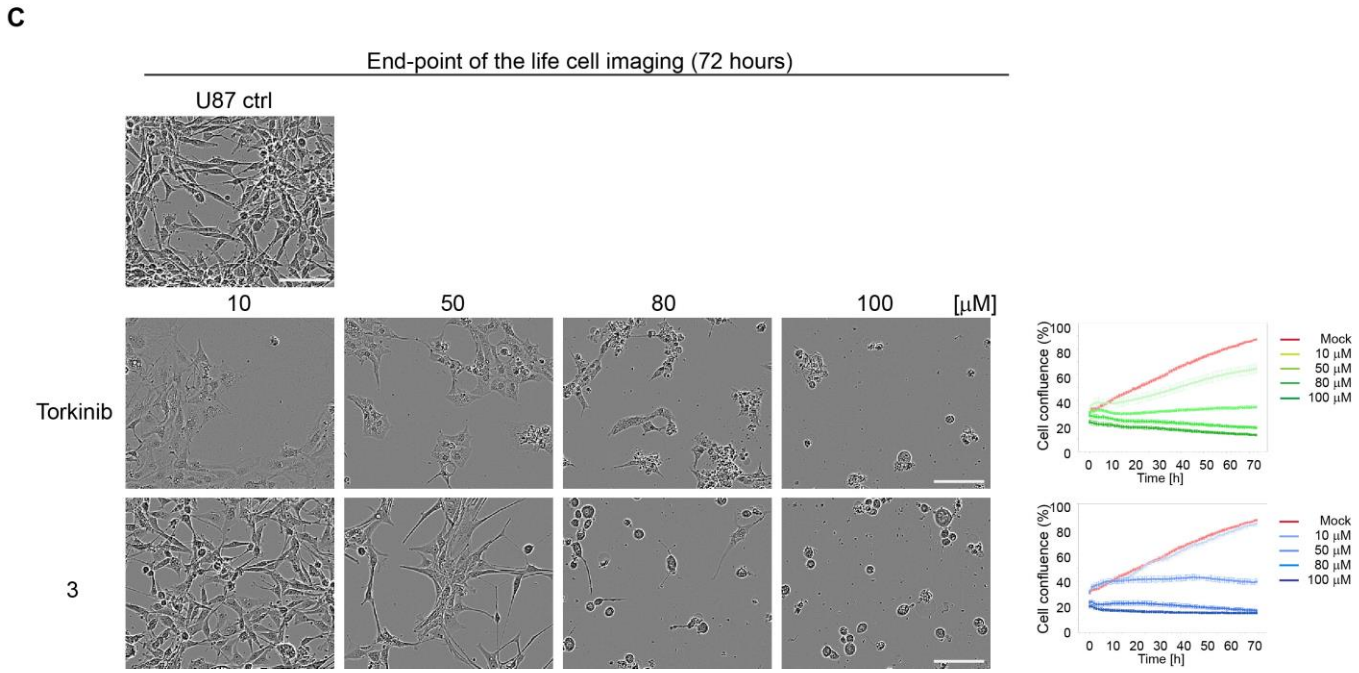

Figure S2. Time-lapse microscopy of new compounds using Incucyte SX1 platform. Proliferating human immortalized retinal pigment epithelium RPE-1 (**A**) glioblastoma U373 (**B**) and U87 (**C**) cells were exposed to compounds **1** – **7** in a concentration range of 0 – 100 μM for 72 h. Torkinib was used as a reference compound. Images were acquired every 60 min. Note that the first image was acquired 30 min (corresponding to 0 hr in graph plots) after adding compounds. Graphs plot the record of changes in cell confluence during the treatment. Average cell confluence and standard error from four images are shown. Bar, 100 μm.

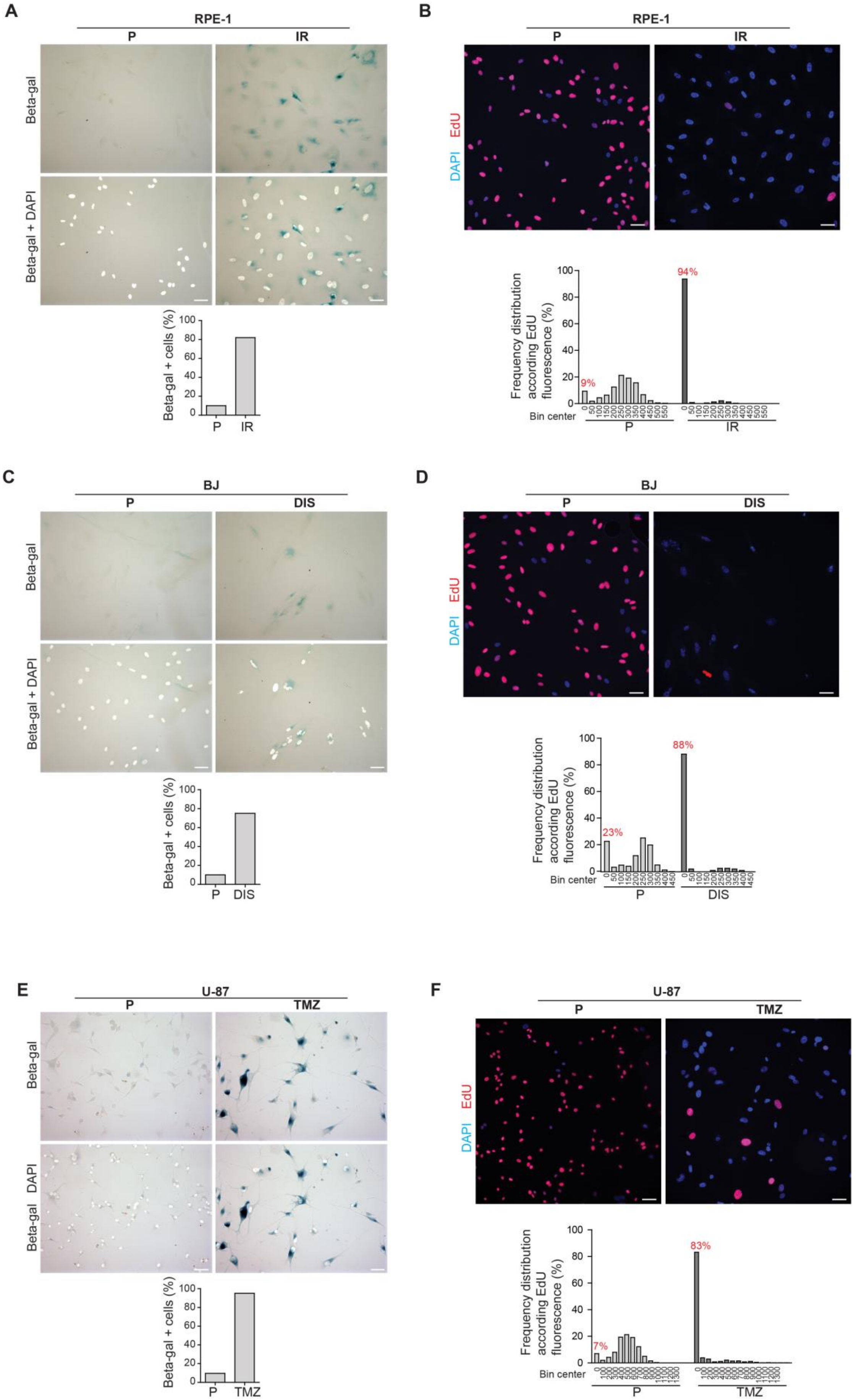
A
RPE-1
P
IR
Beta-gal
Beta-gal + DAPI
Beta-gal + cells (%)
100
80
60
40
20
0
P
IR
B
RPE-1
P
IR
DAPI EdU
Frequency distribution according EdU fluorescence (%)
100
80
60
40
20
0
9%
94%
Bin center
0
50
100
150
200
250
300
350
400
450
500
550
P
IR
C
BJ
P
DIS
Beta-gal
Beta-gal + DAPI
Beta-gal + cells (%)
P
DIS
D
BJ
P
DIS
DAPI EdU
Frequency distribution according EdU fluorescence (%)
23%
88%
Bin center
P
DIS
E
U-87
P
TMZ
Beta-gal
Beta-gal DAPI
Beta-gal + cells (%)
P
TMZ
F
U-87
P
TMZ
DAPI EdU
Frequency distribution according EdU fluorescence (%)
7%
83%
Bin center
P
TMZ

G

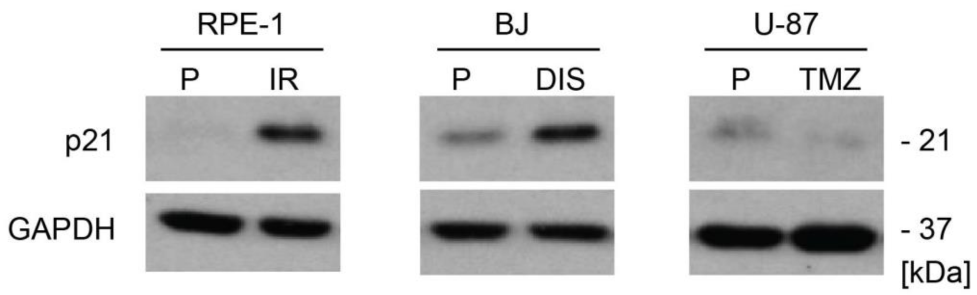

Figure S3. Determination of cellular senescence. Senescence-associated *β*-galactosidase activity in *γ*-irradiated senescent RPE-1 (**A**), docetaxel-induced BJ (**C**), and temozolomide-(TMZ) induced U87 (**E**) cells. Proliferating cells were used as controls. Cell nuclei were stained by DAPI (upper row; white color). The percentage of *β*-galactosidase-positive cells in proliferating and senescent populations was plotted (n > 100). DNA replication activity was detected by EdU incorporation (red color) in *γ*-irradiated senescent RPE-1 (**B**), docetaxel-induced BJ (**D**), and TMZ-induced U-87 (**F**) cells. Proliferating cells were used as controls. Cell nuclei were stained by DAPI (blue color). The percentage of EdU-positive cells was plotted (n > 100). Bar, 50 µm. The level of p21waf1 (p21) was detected by immunoblotting in *γ*-irradiated senescent RPE-1 (**A**), docetaxel-induced BJ (**C**), and TMZ-induced U-87 (**E**) cells. Proliferating cells were used as controls. GAPDH was used as a loading control.

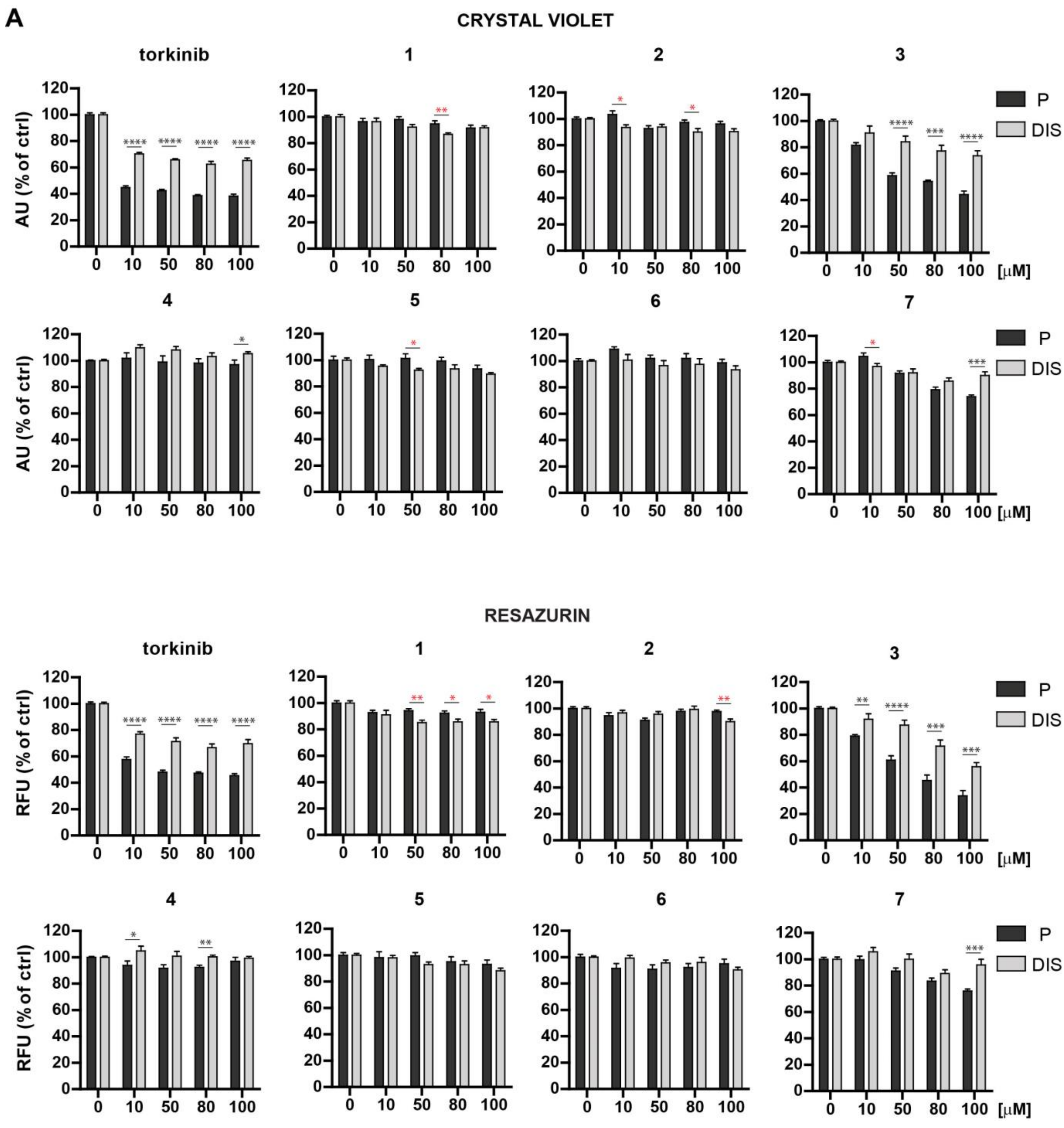
A
CRYSTAL VIOLET
torkinib
1
2
3
4
5
6
7
AU (% of ctrl)
P
DIS
[μM]
RESAZURIN
RFU (% of ctrl)

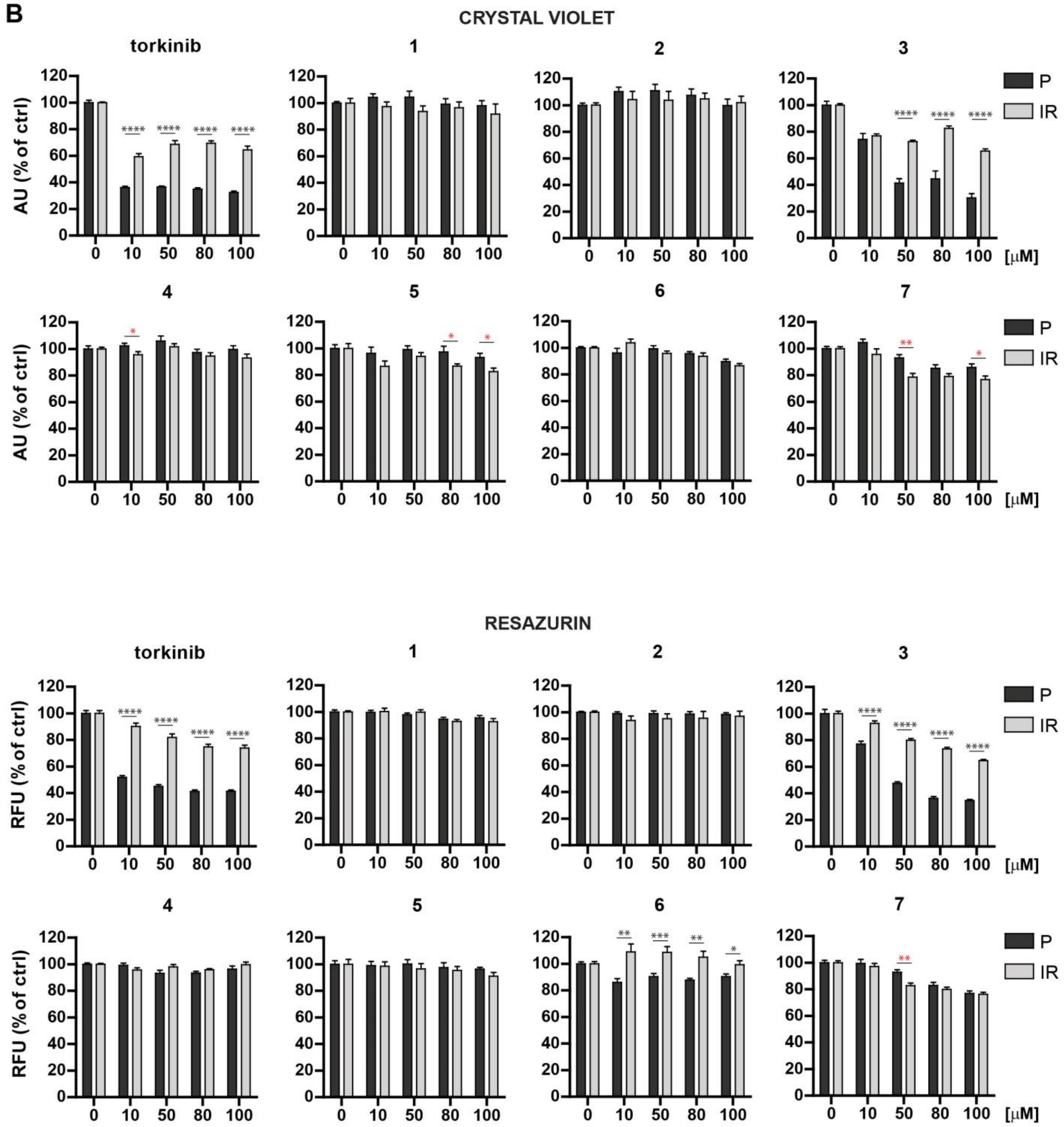

Figure S4. Analysis of selective senolytic effect of new compounds. Data referred to Figure 4 and 6. Proliferating and senescent human BJ fibroblasts (**A**) and immortalized retinal pigment epithelium RPE-1 (**B**) cells were exposed to compounds **1** – **7** in a concentration range of 0 – 100 μM for 24 h and assayed by crystal violet and resazurin assays. Torkinib was used as a reference compound. Human proliferating BJ (P-BJ) fibroblasts brought to docetaxel-induced senescence (DIS-BJ), and human immortalized retinal pigment epithelium proliferating RPE-1 (P-RPE-1) cells set to ionizing radiation-induced senescence (IR-RPE-1) were used. The experiment was performed in triplicate.

Data were normalized to untreated cells and plotted as mean ± SEM. Student's t-test, ****, $p < 0.0001$; ***, $p < 0.001$; **, $p < 0.01$; *, $p < 0.05$. Red stars indicate conditions with a statistically significant selective senolytic effect (i.e., higher toxicity to senescent than proliferating cells). Black stars indicate conditions where proliferating cells were significantly more affected than senescent cells.

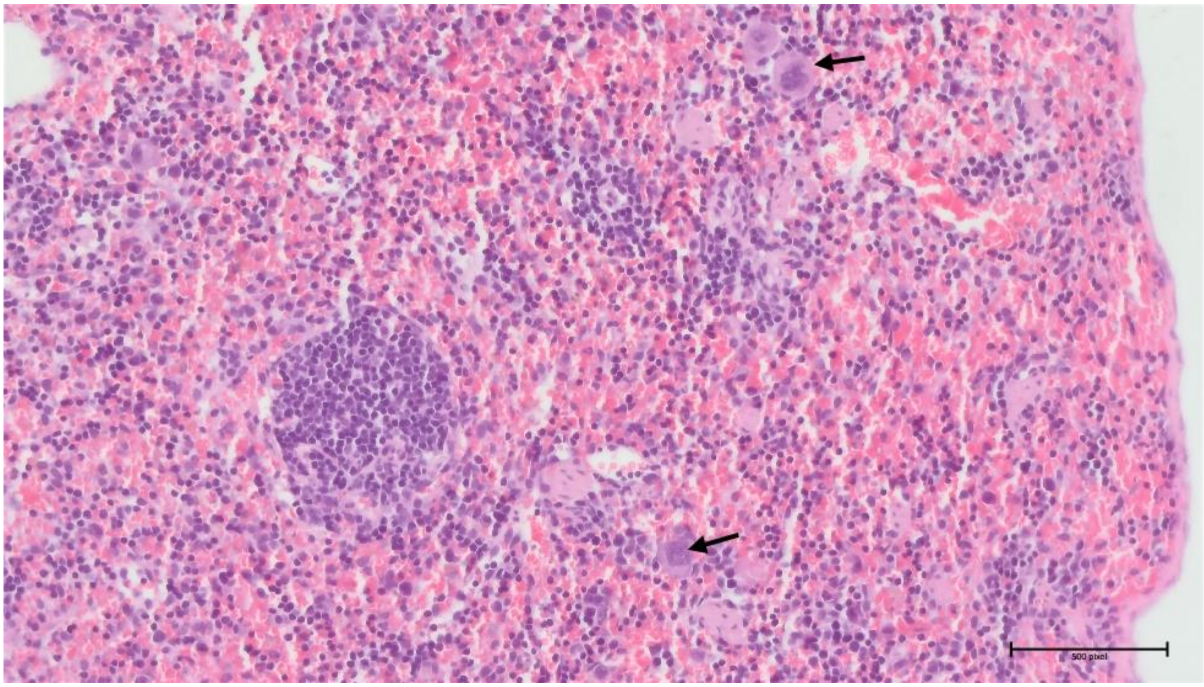

Figure S5. Group **3**/25 – spleen (HE, 100×): mild extramedullar hematopoiesis (arrow)

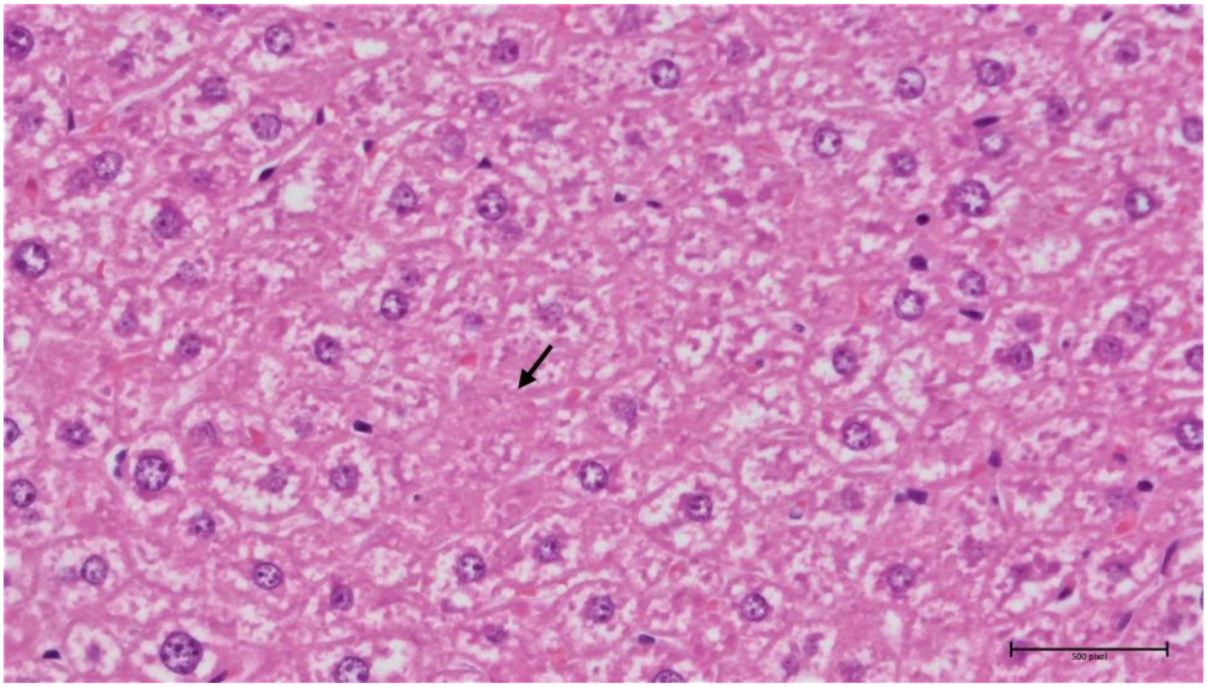

Figure S6. Group **3**/50 – liver (HE, 400×): rare incipient centrilobular necrosis (arrow)
